# ANN-assisted analysis of Hall-MHD Casson blood–gold nanofluid flow over a stretching sheet

**DOI:** 10.1186/s11671-026-05044-0

**Published:** 2026-11-10

**Authors:** Santosh Kumar Choudhary, Kaushik Dehingia, Rana Muhammad Zulqarnaian, Alhanouf Alburaikan, Hamiden Abd El-Wahed Khalifa

**Affiliations:** 1 Manipal Institute of Technology, Manipal Academy of Higher Education, Manipal, India; 2 Department of Mathematics, Sonari College, Sonari, 785690 Assam, India; 3 Research Center of Applied Mathematics, Khazar University, 1096 Baku, Azerbaijan; 4 Department of Mathematics, Saveetha School of Engineering, SIMATS Thandalam, Chennai, 602105 Tamilnadu India; 5 Department of Mathematics, College of Science, Qassim University, Buraydah, 51452 Saudi Arabia; 6 Department of Operations and Management Research, Faculty of Graduate Studies for Statistical Research, Cairo University, Giza, 12613 Egypt

**Keywords:** Casson nanofluid, Nanoparticles, Hall current, Thermal radiation, Stretching sheet, Artificial neural network, Levenberg-Marquardt algorithm, Lobatto IIIA collocation

## Abstract

This study develops a validated numerical-neural framework for the steady boundary-layer flow of a Casson blood-gold (Au) nanofluid over a linearly stretching sheet. The model includes a transverse magnetic field with Hall current, Rosseland thermal radiation, Brownian motion, thermophoresis, internal heat generation and an Arrhenius-type chemical reaction. The mixture properties are computed with the Tiwari-Das model, while Buongiorno's slip mechanisms govern the local nanoparticle concentration. Similarity variables reduce the equations to a coupled nonlinear boundary-value problem, which is solved by fourth-order Lobatto IIIA collocation with residual control (the bvp4c algorithm). The solver reproduces the benchmark data of Khan and Pop to four decimal places and an exact Casson-MHD-nanofluid solution to within ${10}^{-6}$. The numerical solutions (17 710 samples in ten scenarios) are used to train single-hidden-layer (2-12-1) networks with the Levenberg-Marquardt (LM) algorithm. The networks reach test mean-squared errors of ${10}^{-11}$-${10}^{-8}$, regression coefficients $R\mathrm{ > 0.99999}$ and maximum absolute errors below $1.1\times {10}^{-3}$ at unseen parameter values; for the same network, LM gives errors two to five orders of magnitude lower than Adam or stochastic gradient descent. Adding 4% gold to blood raises the heat-transfer rate by 8.0% and lowers the streamwise wall shear by 17.9%, because the gold loading strengthens the thermal buoyancy. Increasing the magnetic parameter from 0.5 to 3 raises the streamwise wall shear by 86.5%, thermal radiation (*R*: 0.1→1.5) doubles the wall heat flux (+112%), and the chemical reaction (*K*: 0.5→2) raises the Sherwood number by 133%. The work is a fundamental transport model and a fast surrogate for the parametric screening of magnetically controlled blood-nanoparticle heat transfer.

## Introduction

**Fig. 1:**
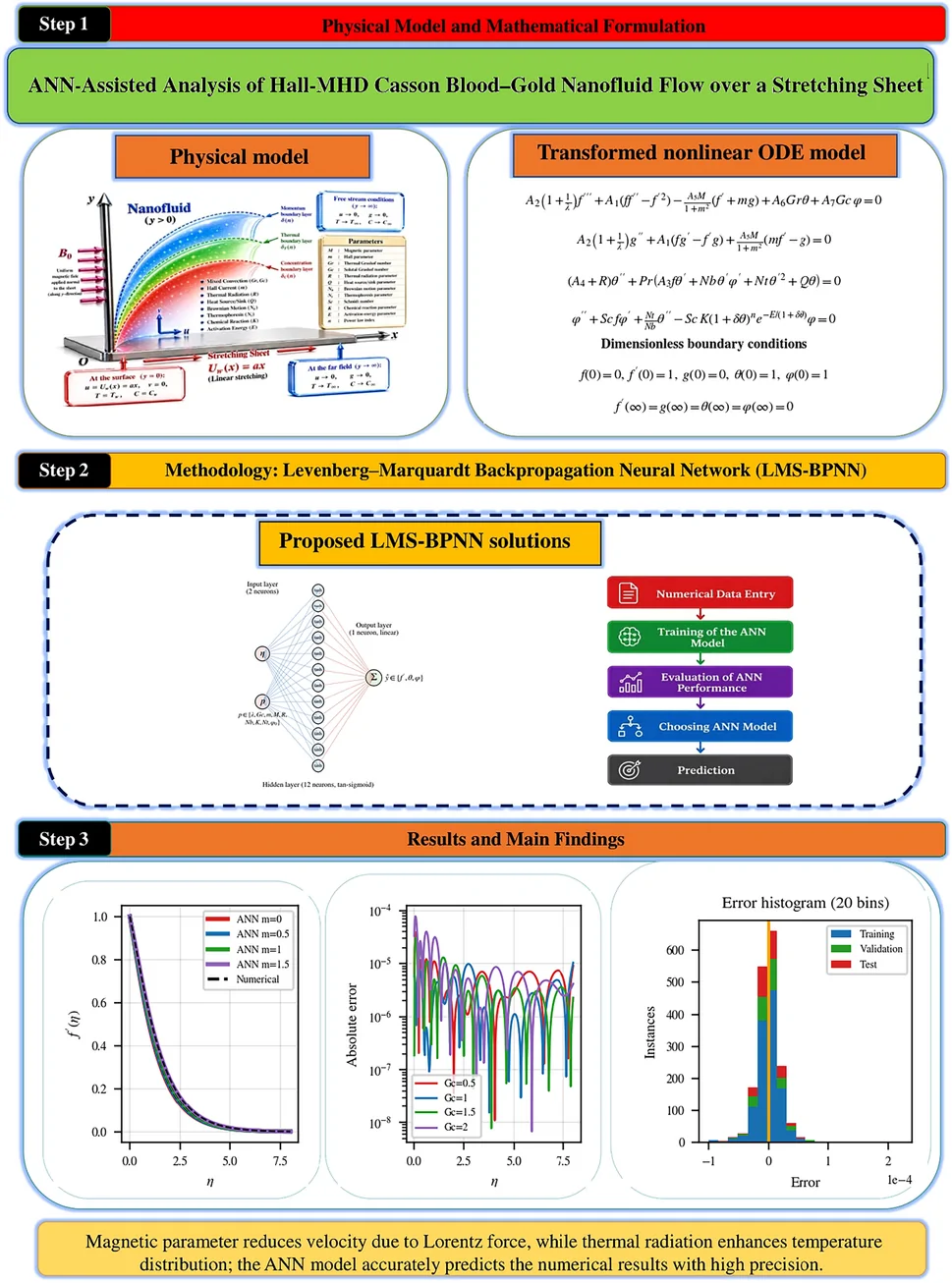
Graphical overview of the study: physical model and transformed equations (Step 1), Levenberg-Marquardt neural-network surrogate (Step 2) and representative results (Step 3)

Fluids whose shear stress does not vary linearly with the strain rate (non-Newtonian fluids) control transport in many industrial, biomedical and engineering systems. Their flow over stretching surfaces has therefore received sustained attention, from polymer extrusion to thermal processing [1, 2]. Among the constitutive models proposed for such fluids, the Casson model [3] captures the yield stress and plug-like core of blood, other biological suspensions, printing inks and some polymer solutions. This explains its wide use in biomedical fluid mechanics [4, 5]. Fluid rheology also governs the performance of engineering devices such as shear-thickening-fluid isolation bearings, magnetorheological clutches, drag- and heat-reducing jets and unsaturated soils [6–9]. Flow and shear effects are likewise central to hydraulic drive control, internal solitary waves in stratified shear currents, shear-stress-induced angiogenesis in vascular chips and sweat transport in wearable membranes [10–13]. They also shape adaptive ferrofluidic robots and the flow-induced vibration of flexible cylinders [14, 15] Fig. 1.

Crane [2] obtained the closed-form solution for the flow induced by a linearly stretching plate, which became the basis of a large literature. Mustafa et al. [16] analysed the unsteady boundary layer of a Casson fluid set in motion by an impulsively started plate, and Mukhopadhyay et al. [4] treated Casson flow over an unsteady stretching surface using the Casson constitutive tensor adopted here. Bhattacharyya [17] studied flow and heat transfer over an exponentially shrinking sheet with suction, and Nadeem et al. [18] extended this configuration to MHD Casson flow, showing that the Lorentz force retards the fluid. Hayat et al. [19] examined mixed-convection stagnation-point Casson flow with convective boundary conditions, and Shehzad et al. [20] reported the effects of mass transfer, chemical reaction and suction on MHD Casson flow over a stretching sheet.

Thermal radiation matters in high-temperature and photothermal applications, and the Rosseland diffusion approximation [21] is the standard model for optically thick media in boundary layers. Cortell [22] showed that radiation thickens the thermal boundary layer over a nonlinearly stretching sheet. Raza [23] studied radiation and slip in MHD stagnation-point Casson flow over a convective stretching sheet. Oyelakin et al. [24] analysed unsteady Casson nanofluid flow over a stretching sheet with radiation and slip, and Alizadeh et al. [25] examined radiation and magnetic effects on mixed convection over a cylinder in a porous medium under local thermal non-equilibrium. Shah et al. [5] obtained fractional solutions for MHD Casson nanofluid flow containing gold nanoparticles, and Hasin et al. [26] studied radiative flow of a vegetable-oil-based Brinkman-type fluid carrying four types of nanoparticles. Earlier, Eldabe et al. [27] examined heat transfer in MHD Casson flow between rotating cylinders, Malik et al. [28] studied Casson nanofluid flow over an exponentially stretching cylinder, and Shah et al. [29] studied radiative carbon-nanotube flow in a rotating Darcy-Forchheimer medium.

For nanofluids, two modelling routes are common. The Tiwari-Das approach [30] treats the suspension as a single phase with effective properties. The Buongiorno model [31] resolves the nanoparticle concentration through Brownian diffusion and thermophoresis, and Khan and Pop [32] applied it to the stretching-sheet boundary layer. Recent studies of related non-Newtonian and nanofluid flows include gyrotactic microorganisms in a Jeffrey nanofluid with the Buongiorno model and Ohmic heating [33], and chemically reactive Jeffrey nanomaterials [34]. Others cover Cross fluids with Arrhenius activation energy [35], thermophoretic and Brownian transport in MHD Hiemenz flow [36], and Oldroyd-B nanofluids with Cattaneo-Christov fluxes in a rotating frame [37]. Curved and rotating geometries include GO-Co_3_O_4_ Homann flow over a biaxially stretching surface [38], Ag-TiO_2_ flow over an inclined stretching surface [39] and viscoelastic Homann stagnation flow with radiation [40]. Further examples are engine-oil-CNT flow on a rotating cylinder [41], entropy generation in Homann-Agrawal flows with logarithmic spiraling [42], and Reiner-Rivlin and Walter-B fluids with modified Cattaneo-Christov fluxes [43, 44]. The Hall current, which arises from the generalized Ohm's law when the electron cyclotron frequency is not small [45], induces a cross flow. Its effect on MHD free convection with mass transfer was quantified by Aboeldahab and Elbarbary [46], and on mixed-convection nanofluid flow over a stretching sheet by Ibrahim and Anbessa [47]. Sadiya et al. [48] recently combined Hall and cross-diffusion effects in a rotating ternary nanofluid. Casson ternary hybrid nanofluids with fractional derivatives were optimized by Saba et al. [49]. Parida et al. [50] examined entropy in unsteady electromagnetic Powell-Eyring hybrid nanofluids for solar water heating, and Dharmaiah et al. [51] studied rotating non-uniform surfaces with Coriolis forces. Daz Palencia et al. [52] used response-surface methodology on radiative hybrid nanofluids.

In parallel, artificial neural networks (ANNs) have become an efficient data-driven complement to numerical solvers. Once trained, they give almost instantaneous predictions over a continuous parameter space. They capture strongly nonlinear input-output maps without assuming a functional form, and they provide smooth interpolants that can be differentiated or embedded in optimization and control loops. The Levenberg-Marquardt (LM) algorithm [53, 54] is the usual training choice for small regression networks. Cheema et al. [55] used LM networks for a nonlinear epidemic system, and Sabir et al. [56] combined ANNs with particle-swarm optimization for singular Emden-Fowler equations. Raja et al. [57] used Bayesian-regularized networks for three-dimensional hybrid nanofluid flow, while Shoaib et al. [58] solved rotating MHD hybrid nanofluid flow with the Lobatto IIIA (bvp4c) method. LM-trained networks have recently reproduced bvp4c solutions for entropy-optimized Jeffrey nanofluids [59] and radiative second-grade fluids [60]. They have also modelled magneto-radiative third-grade fluids with homogeneous-heterogeneous reactions [61] and radiative hybrid nanofluids with Forchheimer resistance [62]. In haemodynamics, LM networks and *K*-fold cross-validation were applied to fractional Jeffrey ternary and trihybrid nanofluids in stenosed arteries [63, 64]. Multi-layer networks were applied to hydride-based ternary nanofluids in a spinning-disk reactor with a Casson parameter [65]. Other data-driven methods include comparative machine-learning models for Marangoni convection [66], adaptive neuro-fuzzy inference with particle-swarm optimization for von Kármán flow [67], deep networks for blood-based penta-hybrid nanofluids in fins [68] and ANN modelling of rotary flow around a permeable sphere [69]. Shamshuddin et al. used ANN-assisted modelling to estimate the transport of a nanostructured Maxwell fluid over an elongated convective sheet with variable thermal conductivity [70], and studied radiative Williamson nanofluid flow over a Riga plate for the thermal regulation of high-power electronic systems [71].

### Research gap and base framework

The present model builds on the Buongiorno-type stretching-sheet formulation of Khan and Pop [32] and on the Hall-current nanofluid model of Ibrahim and Anbessa [47]. It extends them in three ways: (i) Casson rheology with blood as the base fluid; (ii) blood-gold effective properties (density, viscosity, heat capacity, thermal and electrical conductivity, and buoyancy) that enter every transformed equation through the ratios ${A}_{1}-{A}_{7}$; and (iii) Rosseland radiation, internal heat generation and an Arrhenius reaction. The literature above either studies Casson or blood-based flows without the combined Hall-radiation-reaction physics, or trains ANNs on a few fixed parameter values without testing their interpolation to unseen values, cross-validation or the choice of optimizer. To our knowledge, no study has examined how the gold loading changes wall shear, heat and mass transfer in a Hall-MHD Casson blood flow, or tested a neural surrogate of this system with these generalization checks.

### Novelty

The main contributions are:

1. a consistent Casson-Hall-radiative blood-Au nanofluid model that combines Tiwari-Das effective properties with Buongiorno slip transport, written in vector form, reduced to a verified similarity system, and validated against published benchmarks and an exact nanofluid solution;
2. a quantitative account of the gold loading, which strengthens the thermal buoyancy of blood (the ratio ${A}_{6}$ rises from 1 to 6.7 at ${\varphi }_{0}\mathrm{=0.04}$) and thereby lowers the streamwise wall shear while raising the Nusselt number;
3. a reproducible LM surrogate trained on dense parameter grids, with hidden-neuron selection, 5-fold cross-validation, interpolation to unseen parameter levels and a direct comparison with Adam and SGD.

### Research questions

The study addresses: (RQ1) How do the Casson parameter, Hall current, magnetic field and buoyancy reshape the primary and secondary velocity fields of blood-Au flow? (RQ2) How strongly do radiation, Brownian motion, thermophoresis and chemical reaction control the wall heat and mass fluxes? (RQ3) By how much does adding gold nanoparticles change the skin friction and Nusselt number relative to pure blood? (RQ4) Can a compact LM-trained network reproduce the numerical solutions accurately at parameter values it has not seen, and is LM preferable to first-order optimizers for this task?

The paper is organized as follows. Section 2 presents the model, Section 3 the numerical procedure and its validation, Section 4 the ANN framework, Section 5 the results, and Section 6 the limitations, followed by the conclusions.

## Mathematical formulation

### Physical model, coordinate system and assumptions

**Fig. 2:**
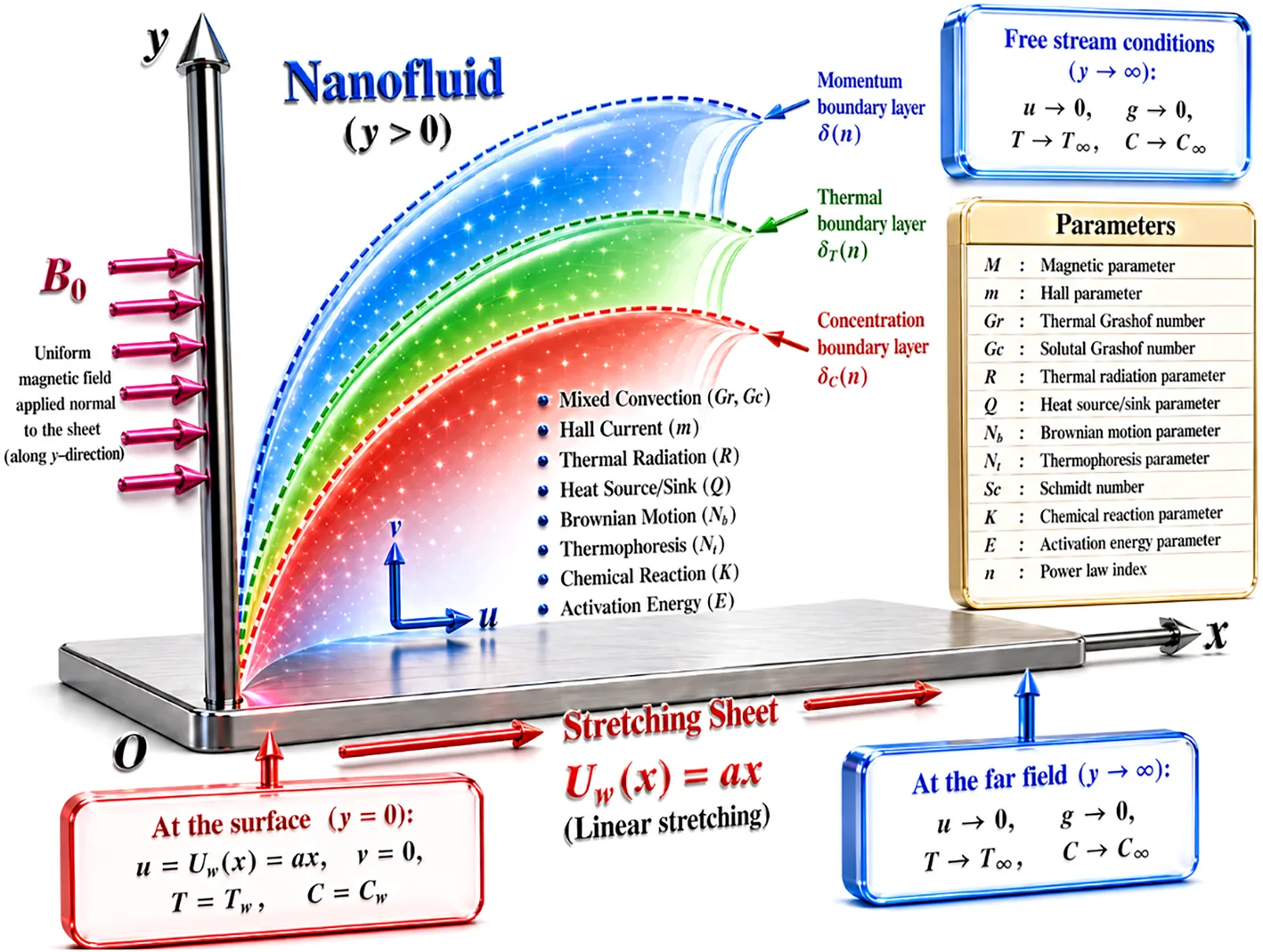
Schematic representation of the physical model and coordinate system

Figure 2 shows the configuration. An impermeable sheet coincides with the plane $$y\mathrm{=0}$$ and is stretched along *x* with velocity $${U}_{w}=ax$$$$(a\mathrm{ > 0})$$, with the origin fixed at the slot from which the sheet emerges. The fluid occupies $$y\mathrm{ > 0}$$ and the *z* axis completes a right-handed system; all quantities are independent of *z*. A uniform magnetic induction $${\bf{B}}\mathrm{=(0,}{B}_{0}\mathrm{,0)}$$ is applied normal to the sheet. The main assumptions are:

(A1) Steady, laminar, incompressible flow of a Casson blood-Au nanofluid; the plastic viscosity is augmented by the particles through the Brinkman relation.

(A2) Gold nanoparticles are spherical, well dispersed and dilute $({\varphi }_{0}\le 0.04)$, in thermal equilibrium and without slip relative to blood at the level of the bulk properties (Tiwari-Das); the small local deviations of their concentration *C* are transported by Brownian diffusion and thermophoresis (Buongiorno).

(A3) The magnetic Reynolds numbser is small, so the induced field and the Hall-induced field are negligible relative to ${B}_{0}$; no external electric field is applied $({\bf{E}}={\bf{0}})$; the Hall effect is retained through the generalized Ohm's law, while ion slip is neglected.

(A4) Radiation is described by the Rosseland approximation for an optically thick, absorbing-emitting medium, linearized for small temperature differences.

(A5) Buoyancy obeys the Boussinesq approximation; viscous dissipation, Joule heating and Soret/Dufour cross-diffusion are neglected.

(A6) The nanoparticle-concentration equation includes a first-order, temperature-activated (Arrhenius) consumption term that represents a generic particle-binding or reaction sink.

(A7) The wall temperature ${T}_{w}$ and concentration ${C}_{w}$ are uniform and exceed the ambient values ${T}_{\infty }$ and ${C}_{\infty }$; the ambient fluid is quiescent.

### Governing equations in vector form

With velocity ${\bf{V}}=(u,v,w)$, the conservation laws for the nanofluid read [4, 30, 31, 45]

1 $$ \nabla \cdot {\bf{V}}\mathrm{=0,} $$

2 $$ \begin{array}{c}{\rho }_{nf}({\bf{V}}\cdot \nabla ){\bf{V}}=-\nabla p+\nabla \cdot {\bf{S}}+{\bf{J}}\times {\bf{B}}\\ +[{(\rho {\beta }_{T})}_{nf}(T-{T}_{\infty })+{(\rho {\beta }_{C})}_{nf}(C-{C}_{\infty })]{{\bf{g}}}_{c},\end{array} $$

3 $$ \begin{array}{c}{(\rho {c}_{p})}_{nf}({\bf{V}}\cdot \nabla T)={k}_{nf}{\nabla }^{2}T-\nabla \cdot {{\bf{q}}}_{r}+{Q}_{0}(T-{T}_{\infty })\\ +{(\rho c)}_{p}[{D}_{B}\nabla C\cdot \nabla T+\frac{{D}_{T}}{{T}_{\infty }}\nabla T\cdot \nabla T],\end{array} $$

4 $$ {\bf{V}}\cdot \nabla C={D}_{B}{\nabla }^{2}C+\frac{{D}_{T}}{{T}_{\infty }}{\nabla }^{2}T-{k}_{r}^{2}(C-{C}_{\infty }){\left(\frac{T}{{T}_{\infty }}\right)}^{n}\exp \left(-\frac{{E}_{a}}{\kappa T}\right)\mathrm{}. $$

The Casson extra stress is [3, 4]

5 $$ \begin{array}{cc}{S}_{ij}=\Bigg\{\begin{array}{cc}2({\mu }_{B}+{p}_{y}/\sqrt{2\pi }){e}_{ij}, & \pi > {\pi }_{c},\\ 2({\mu }_{B}+{p}_{y}/\sqrt{2{\pi }_{c}}){e}_{ij}, & \pi < {\pi }_{c},\end{array} & \pi ={e}_{ij}{e}_{ij},\end{array} $$

so that in the sheared boundary layer the effective viscosity is $\mu \mathrm{(1}+\mathrm{1/}\lambda )$, with Casson parameter $\lambda ={\mu }_{B}\sqrt{2{\pi }_{c}}/{p}_{y}$. The current density follows from the generalized Ohm's law with Hall effect [45, 46]

6 $$ \begin{array}{cc}{\bf{J}}+\frac{m}{{B}_{0}}\,{\bf{J}}\times {\bf{B}}={\sigma }_{nf}({\bf{E}}+{\bf{V}}\times {\bf{B}}), & m={\omega }_{e}{\tau }_{e}\mathrm{}.\end{array} $$

With ${\bf{E}}={\bf{0}}$ and ${\bf{B}}\mathrm{=(0,}{B}_{0}\mathrm{,0)}$, Eq. (6) gives ${J}_{x}={\sigma }_{nf}{B}_{0}(mu-w\mathrm{)/(1}+{m}^{2})$ and ${J}_{z}={\sigma }_{nf}{B}_{0}(u+mw\mathrm{)/(1}+{m}^{2})$, so the Lorentz force is

7 $$ {\bf{J}}\times {\bf{B}}=\frac{{\sigma }_{nf}{B}_{0}^{2}}{1+{m}^{2}}(-(u+mw),\,\mathrm{0,}\,mu-w)\mathrm{}. $$

### Boundary-layer equations

Under the usual boundary-layer ordering $(\partial /\partial x\ll \partial /\partial y,\,v\ll u)$ and a zero pressure gradient imposed by the quiescent ambient fluid, Eqs. (1)-(7) reduce to [4, 32, 47]

8 $$ \frac{\partial u}{\partial x}+\frac{\partial v}{\partial y}\mathrm{=0,} $$

9 $$ \begin{array}{c}{\rho }_{nf}\left(u\frac{\partial u}{\partial x}+v\frac{\partial u}{\partial y}\right)={\mu }_{nf}\left(1+\frac{1}{\lambda }\right)\frac{{\partial }^{2}u}{\partial {y}^{2}}-\frac{{\sigma }_{nf}{B}_{0}^{2}}{1+{m}^{2}}(u+mw)\\ +{(\rho {\beta }_{T})}_{nf}{g}_{c}(T-{T}_{\infty })+{(\rho {\beta }_{C})}_{nf}{g}_{c}(C-{C}_{\infty }),\end{array} $$

10 $$ {\rho }_{nf}\left(u\frac{\partial w}{\partial x}+v\frac{\partial w}{\partial y}\right)={\mu }_{nf}\left(1+\frac{1}{\lambda }\right)\frac{{\partial }^{2}w}{\partial {y}^{2}}+\frac{{\sigma }_{nf}{B}_{0}^{2}}{1+{m}^{2}}(mu-w), $$

11 $$ \begin{array}{ll}{(\rho {c}_{p})}_{nf}\left(u\frac{\partial T}{\partial x}+v\frac{\partial T}{\partial y}\right)= & {k}_{nf}\frac{{\partial }^{2}T}{\partial {y}^{2}}-\frac{\partial {q}_{r}}{\partial y}+{Q}_{0}(T-{T}_{\infty })\\ & +{(\rho c)}_{p}\left[{D}_{B}\frac{\partial C}{\partial y}\frac{\partial T}{\partial y}+\frac{{D}_{T}}{{T}_{\infty }}{(\frac{\partial T}{\partial y})}^{2}\right],\end{array} $$

12 $$ u\frac{\partial C}{\partial x}+v\frac{\partial C}{\partial y}={D}_{B}\frac{{\partial }^{2}C}{\partial {y}^{2}}+\frac{{D}_{T}}{{T}_{\infty }}\frac{{\partial }^{2}T}{\partial {y}^{2}}-{k}_{r}^{2}(C-{C}_{\infty }){\left(\frac{T}{{T}_{\infty }}\right)}^{n}\exp \left(\frac{-{E}_{a}}{\kappa T}\right), $$

subject to

13 $$ \begin{array}{c}\begin{array}{cccccc}u=ax, & v\mathrm{=0,} & w\mathrm{=0,} & T={T}_{w}, & C={C}_{w} & {\rm{at}}y\mathrm{=0,}\end{array}\\ \begin{array}{ccccc}u\to \mathrm{0,} & w\to \mathrm{0,} & T\to {T}_{\infty }, & C\to {C}_{\infty } & {\rm{as}}y\to \infty \mathrm{}.\end{array}\end{array} $$

#### Physical meaning of the boundary conditions

At the wall, the no-slip condition makes the fluid move with the sheet (*u=ax*, $w\mathrm{=0}$), and the impermeability condition gives $v\mathrm{=0}$. The sheet is held at a uniform temperature ${T}_{w}$ (a heated surface) and concentration ${C}_{w}$ (e.g. a particle-releasing coating). Far from the sheet the fluid is undisturbed and at rest with ambient temperature and concentration. These are the standard conditions for the Crane problem and its nanofluid extensions [2, 32, 47].

Under the Rosseland approximation [21], the radiative flux is

14 $$ {q}_{r}=-\frac{4{\sigma }_{1}}{3{k}^{\ast }}\frac{\partial {T}^{4}}{\partial y}, $$

where ${k}^{\ast }$ is the Rosseland mean absorption coefficient and ${\sigma }_{1}$ the Stefan-Boltzmann constant. Because the temperature difference across the layer is small $(\delta \ll 1)$, ${T}^{4}$ is expanded about ${T}_{\infty }$ and higher-order terms are discarded:

15 $$ {T}^{4}\approx 4{T}_{\infty }^{3}T-3{T}_{\infty }^{4}, $$

so that $-\partial {q}_{r}/\partial y\mathrm{=(16}{\sigma }_{1}{T}_{\infty }^{3}\mathrm{/3}{k}^{\ast })\,{\partial }^{2}T/\partial {y}^{2}$ acts as an additional radiative conductivity in Eq. (11).

### Effective properties and the Tiwari-Das/Buongiorno combination

The base fluid is human blood, in which gold nanoparticles are dispersed to form a Casson-type nanofluid. The mixture properties (subscript *nf*) are evaluated at the reference loading ${\varphi }_{0}$ with linear mixing rules for density and heat capacity, the Brinkman relation for viscosity [72], and Maxwell's relations for thermal and electrical conductivity [30, 73]:

16a $$ \begin{array}{cc}{\rho }_{nf}\mathrm{=(1}-{\varphi }_{0}){\rho }_{f}+{\varphi }_{0}{\rho }_{s}, & {(\rho {c}_{p})}_{nf}\mathrm{=(1}-{\varphi }_{0})(\rho {c}_{p}{)}_{f}+{\varphi }_{0}{(\rho {c}_{p})}_{s},\end{array} $$

16b $$ {\mu }_{nf}=\frac{{\mu }_{f}}{{\mathrm{(1}-{\varphi }_{0})}^{2.5}}, $$

16c $$ \frac{{k}_{nf}}{{k}_{f}}=\frac{{k}_{s}+2{k}_{f}-2{\varphi }_{0}({k}_{f}-{k}_{s})}{{k}_{s}+2{k}_{f}+{\varphi }_{0}({k}_{f}-{k}_{s})}, $$

16d $$ \frac{{\sigma }_{nf}}{{\sigma }_{f}}\mathrm{=1}+\frac{3({\sigma }_{s}/{\sigma }_{f}-1){\varphi }_{0}}{({\sigma }_{s}/{\sigma }_{f}+2)-({\sigma }_{s}/{\sigma }_{f}-1){\varphi }_{0}}, $$

16e $$ {(\rho {\beta }_{T})}_{nf}\mathrm{=(1}-{\varphi }_{0})(\rho {\beta }_{T}{)}_{f}+{\varphi }_{0}{(\rho {\beta }_{T})}_{s},\,{(\rho {\beta }_{C})}_{nf}={\rho }_{nf}{\beta }_{C}, $$

with the property values given in Table 1. The solutal expansion coefficient ${\beta }_{C}$ is taken to be the same for the carrier and the mixture, so that ${(\rho {\beta }_{C})}_{nf}/(\rho {\beta }_{C}{)}_{f}={\rho }_{nf}/{\rho }_{f}$. The property ratios are

17 $$ \begin{array}{c}{A}_{1}=\frac{{\rho }_{nf}}{{\rho }_{f}},\,{A}_{2}=\frac{{\mu }_{nf}}{{\mu }_{f}},\,{A}_{3}=\frac{{(\rho {c}_{p})}_{nf}}{{(\rho {c}_{p})}_{f}},\,{A}_{4}=\frac{{k}_{nf}}{{k}_{f}},\\ {A}_{5}=\frac{{\sigma }_{nf}}{{\sigma }_{f}},\,{A}_{6}=\frac{{(\rho {\beta }_{T})}_{nf}}{{(\rho {\beta }_{T})}_{f}},\,{A}_{7}=\frac{{(\rho {\beta }_{C})}_{nf}}{{(\rho {\beta }_{C})}_{f}}={A}_{1}\mathrm{}.\end{array} $$

**Table 1:** Thermophysical properties of blood (base fluid) and gold (Au) nanoparticles used in this study (values as commonly adopted for blood-gold nanofluids, e.g. [5])

Property	Blood (*f*)	Gold, Au (*s*)
Density, *ρ * (kg m^−3^)	1050	19300
Specific heat, ${c}_{p}$ (J kg^−1^ K^−1^)	3617	129
Thermal conductivity, *k* (W m^−1^ K^−1^)	0.52	318
Electrical conductivity, *σ * (S m^−1^)	0.8	$4.1\times {10}^{7}$
Thermal expansion coeff., ${\beta }_{T}$ (${10}^{-5}$ K^−1^)	0.18	1.4

At ${\varphi }_{0}\mathrm{=0.02}$ they take the values ${A}_{1}\mathrm{=1.348}$, ${A}_{2}\mathrm{=1.052}$, ${A}_{3}\mathrm{=0.993}$, ${A}_{4}\mathrm{=1.061}$, ${A}_{5}\mathrm{=1.061}$ and ${A}_{6}\mathrm{=3.839}$. The large value of ${A}_{6}$ follows from ${(\rho {\beta }_{T})}_{s}/(\rho {\beta }_{T}{)}_{f}\approx 143$ for the data in Table 1.

#### Applicability of the property model

The Maxwell and Brinkman relations are derived for dilute suspensions of non-interacting spheres. They are appropriate for the low loadings considered here $({\varphi }_{0}\le 0.04)$, but they do not account for particle size, clustering, an interfacial layer or the haematocrit-dependent microstructure of blood [30, 72, 73].

#### Theoretical basis for combining the two approaches

The Tiwari-Das model fixes the *bulk* composition ${\varphi }_{0}$ and hence the transport properties, while Eq. (12) describes the *local* redistribution of nanoparticles by Brownian diffusion and thermophoresis, as in Buongiorno's two-component model [31, 32]. The two descriptions are consistent when the concentration perturbation is small compared with the mean loading, $|C-{C}_{\infty }|\le {C}_{w}-{C}_{\infty }\ll {\varphi }_{0}$. In that case the property variations caused by the redistribution are of second order and can be neglected in the momentum and energy balances, whereas the slip fluxes, which are first order in the concentration and temperature gradients, are retained. The dimensionless $\varphi (\eta )$ therefore measures the normalized local deviation of the nanoparticle concentration, not the loading itself. This hybrid description has been used in several nanofluid boundary-layer studies (e.g. [33, 36, 37]), and its restriction to small perturbations is listed among the limitations in Section 6.

#### Magnetic interaction in a blood-gold suspension

Gold is diamagnetic, not ferromagnetic, so no magnetization (Kelvin) body force is included. The magnetic coupling in the model arises solely from the Lorentz force on the *electric current* carried by the conducting mixture. Blood is an electrolyte with ${\sigma }_{f}\approx 0.8$ S m^−1^, and the dispersed gold spheres raise the effective conductivity through the Maxwell relation in Eq. (16); for ${\sigma }_{s}\gg {\sigma }_{f}$ this gives ${A}_{5}\approx 1+3{\varphi }_{0}\mathrm{/(1}-{\varphi }_{0})$, i.e. about 6% at ${\varphi }_{0}\mathrm{=0.02}$. For a realistic parameter range, $M={\sigma }_{f}{B}_{0}^{2}/({\rho }_{f}a)$ gives ${\sigma }_{f}{B}_{0}^{2}/{\rho }_{f}\approx 1.7\times {10}^{-3}$ s^−1^ for a clinical field of ${B}_{0}\mathrm{=1.5}$ T and $6.9\times {10}^{-3}$ s^−1^ for ${B}_{0}\mathrm{=3}$ T. The range $M\mathrm{=0.5}-3$ used here therefore corresponds to slow surface deformation rates, $a\sim {10}^{-3}-{10}^{-2}$ s^−1^, under strong fields; at larger physiological strain rates *M≪ 1* and magnetic effects on blood are weak. Similarly, the Hall parameter $m={\omega }_{e}{\tau }_{e}$ of an electrolyte is small, and the values $m\mathrm{=0}-1.5$ are included, following the MHD literature [46, 47], to map the mathematical response of the model rather than a specific physiological condition.

### Similarity transformation

We introduce the stream function $\psi =\sqrt{a{\nu }_{f}}\,xf(\eta )$ with $u=\partial \psi /\partial y$ and $v=-\partial \psi /\partial x$, together with [2, 32]

18 $$ \begin{array}{c}\eta =\sqrt{\frac{a}{{\nu }_{f}}}\,y,\,u=axf{\prime} (\eta ),\,v=-\sqrt{a{\nu }_{f}}\,f(\eta ),\,w=axg(\eta ),\\ \theta (\eta )=\frac{T-{T}_{\infty }}{{T}_{w}-{T}_{\infty }},\,\varphi (\eta )=\frac{C-{C}_{\infty }}{{C}_{w}-{C}_{\infty }}\mathrm{}.\end{array} $$

Equation (8) is then satisfied identically, since $\partial u/\partial x+\partial v/\partial y=af{\prime} -af{\prime} \mathrm{=0}$. The similarity form is justified because the imposed wall velocity is proportional to *x* and the flow has no other length scale: the self-similar profiles make the convective terms scale as ${a}^{2}x$, like the viscous, Lorentz and buoyancy terms. With the linearized radiative flux, substituting Eq. (18) into Eqs. (9)-(12) and dividing by ${\rho }_{f}{a}^{2}x$, $a{(\rho {c}_{p})}_{f}({T}_{w}-{T}_{\infty })/\,Pr$ and $a({C}_{w}-{C}_{\infty })/Sc$, respectively, gives

19 $$ {A}_{2}\left(1+\frac{1}{\lambda }\right)f{\prime} {\prime} {\prime} +{A}_{1}(ff{\prime} {\prime} -{f{\prime} }^{2})-\frac{{A}_{5}M}{1+{m}^{2}}(f{\prime} +mg)+{A}_{6}\,Gr\,\theta +{A}_{7}\,Gc\,\varphi \mathrm{=0,} $$

20 $$ {A}_{2}\left(1+\frac{1}{\lambda }\right)g{\prime} {\prime} +{A}_{1}(fg{\prime} -f{\prime} g)+\frac{{A}_{5}M}{1+{m}^{2}}(mf{\prime} -g)\mathrm{=0,} $$

21 $$ ({A}_{4}+R)\,\theta {\prime} {\prime} +Pr({A}_{3}f\theta {\prime} +Nb\,\theta {\prime} \varphi {\prime} +Nt\,{\theta {\prime} }^{2}+Q\theta )\mathrm{=0,} $$

22 $$ \varphi {\prime} {\prime} +Sc\,f\varphi {\prime} +\frac{Nt}{Nb}\,\theta {\prime} {\prime} -Sc\,K{(1+\delta \theta )}^{n}\exp \left(\frac{-E}{1+\delta \theta }\right)\varphi \mathrm{=0,} $$

with

23 $$ \begin{array}{c}f\mathrm{(0)=0,}\,f{\prime} \mathrm{(0)=1,}\,g\mathrm{(0)=0,}\,\theta \mathrm{(0)=1,}\,\varphi \mathrm{(0)=1,}\\ f{\prime} (\eta )\to \mathrm{0,}\,g(\eta )\to \mathrm{0,}\,\theta (\eta )\to \mathrm{0,}\,\varphi (\eta )\to 0\,{\rm{as}}\eta \to \infty \mathrm{}.\end{array} $$

The dimensionless parameters are

24 $$ \begin{array}{c}M=\frac{{\sigma }_{f}{B}_{0}^{2}}{{\rho }_{f}a},\,Gr=\frac{{g}_{c}{\beta }_{T}({T}_{w}-{T}_{\infty })}{{a}^{2}x}=\frac{G{r}_{x}}{R{e}_{x}^{2}},\,Gc=\frac{{g}_{c}{\beta }_{C}({C}_{w}-{C}_{\infty })}{{a}^{2}x}=\frac{G{c}_{x}}{R{e}_{x}^{2}},\\ Pr=\frac{{\nu }_{f}{(\rho {c}_{p})}_{f}}{{k}_{f}},\,R=\frac{16{\sigma }_{1}{T}_{\infty }^{3}}{3{k}^{\ast }{k}_{f}},\,Q=\frac{{Q}_{0}}{a{(\rho {c}_{p})}_{f}},\,Nb=\frac{\tau {D}_{B}({C}_{w}-{C}_{\infty })}{{\nu }_{f}},\\ Nt=\frac{\tau {D}_{T}({T}_{w}-{T}_{\infty })}{{\nu }_{f}{T}_{\infty }},\,\tau =\frac{{(\rho c)}_{p}}{{(\rho {c}_{p})}_{f}},\\ Sc=\frac{{\nu }_{f}}{{D}_{B}},\,K=\frac{{k}_{r}^{2}}{a},\,\delta =\frac{{T}_{w}-{T}_{\infty }}{{T}_{\infty }},\,E=\frac{{E}_{a}}{\kappa {T}_{\infty }},\,R{e}_{x}=\frac{a{x}^{2}}{{\nu }_{f}},\end{array} $$

where $$G{r}_{x}={g}_{c}{\beta }_{T}({T}_{w}-{T}_{\infty }){x}^{3}/{\nu }_{f}^{2}$$ and $$G{c}_{x}={g}_{c}{\beta }_{C}({C}_{w}-{C}_{\infty }){x}^{3}/{\nu }_{f}^{2}$$ are the local Grashof numbers. The buoyancy parameters *Gr* and *Gc* are thus the ratios of buoyancy to inertia (Richardson numbers). For a uniform wall temperature and concentration they vary as $${x}^{-1}$$, so the solutions with buoyancy are *locally* similar: *Gr* and *Gc* are treated as constants at a given station, as is standard in mixed-convection stretching-sheet analyses [47]. Exact similarity for *Gr* and *Gc* would require $${T}_{w}-{T}_{\infty }\propto x$$ and $${C}_{w}-{C}_{\infty }\propto x$$, but this would make *Nb*, *Nt* and *δ * depend on *x*; the local-similarity choice keeps the energy and concentration equations exactly similar. In the concentration equation, the Schmidt number $$Sc={\nu }_{f}/{D}_{B}=Pr\,Le$$$$({\rm{with}}\,Le={\alpha }_{f}/{D}_{B})$$ multiplies the convective and reaction terms. In the limit $${\varphi }_{0}\mathrm{=0}$$, $$\lambda \to \infty $$, $$M=Gr=Gc=R=Q=K\mathrm{=0}$$, Eqs. (19)-(22) reduce exactly to the Khan-Pop system [32].

### Physical quantities of engineering interest

The streamwise and transverse skin-friction coefficients, the local Nusselt number and the local Sherwood number are defined at the sheet ($y\mathrm{=0}$, the only wall in this configuration) as

25 $$ {C}_{fx}=\frac{{\tau }_{wx}}{{\rho }_{f}{U}_{w}^{2}},\,{C}_{fz}=\frac{{\tau }_{wz}}{{\rho }_{f}{U}_{w}^{2}},\,N{u}_{x}=\frac{x\,{q}_{w}}{{k}_{f}({T}_{w}-{T}_{\infty })},\,S{h}_{x}=\frac{x\,{j}_{w}}{{D}_{B}({C}_{w}-{C}_{\infty })}, $$

where

26 $$ \begin{array}{c}{\tau }_{wx}={\mu }_{nf}\left(1+\frac{1}{\lambda }\right){\frac{\partial u}{\partial y}\big|}_{0},\,{\tau }_{wz}={\mu }_{nf}\left(1+\frac{1}{\lambda }\right){\frac{\partial w}{\partial y}\big|}_{0},\\ {q}_{w}=-\left({k}_{nf}+\frac{16{\sigma }_{1}{T}_{\infty }^{3}}{3{k}^{\ast }}\right){\frac{\partial T}{\partial y}\big|}_{0},\,{j}_{w}=-{D}_{B}{\frac{\partial C}{\partial y}\big|}_{0}\mathrm{}.\end{array} $$

In similarity variables,

27 $$ \begin{array}{c}R{e}_{x}^{\mathrm{1/2}}{C}_{fx}={A}_{2}\left(1+\frac{1}{\lambda }\right)f{\prime} {\prime} \mathrm{(0),}\,R{e}_{x}^{\mathrm{1/2}}{C}_{fz}={A}_{2}\left(1+\frac{1}{\lambda }\right)g{\prime} \mathrm{(0),}\\ R{e}_{x}^{-\mathrm{1/2}}N{u}_{x}=-({A}_{4}+R)\,\theta {\prime} \mathrm{(0),}\,R{e}_{x}^{-\mathrm{1/2}}S{h}_{x}=-\varphi {\prime} (0).\end{array} $$

Sign convention: ${\tau }_{wx}\mathrm{ < 0}$ means that the stretching sheet drags the adjacent fluid, so a larger $|{C}_{fx}|$ means a larger force (and power) needed to drive the sheet; $N{u}_{x}\mathrm{ > 0}$ and $S{h}_{x}\mathrm{ > 0}$ denote heat and mass transfer from the sheet to the fluid.

### Parameter values

Table 2 lists the baseline values, the ranges swept and their physical basis. Unless stated otherwise, all figures and tables use the baseline values.

**Table 2:** Dimensionless parameters: baseline values, ranges and physical basis

Parameter	Baseline	Range	Physical basis
*λ *	0.5	0.2-2.0	Casson parameter of blood-like suspensions [4, 18]
*M*	1.0	0.5-3.0	${B}_{0}\mathrm{=1.5}-3$ T with slow stretching, $a\sim {10}^{-3}$-${10}^{-2}$ s^−1^ (Sec. 2.4)
*m*	0.5	0-1.5	Hall parameter, parametric range of [46, 47]
*Gr*, *Gc*	0.5, 0.5	*Gc*: 0.5-2.0	assisting mixed convection, $G{r}_{x}/R{e}_{x}^{2}=O\mathrm{(1)}$
*Pr*	21	-	blood, ${\mu }_{f}\approx 3\times {10}^{-3}$ Pa s, ${c}_{p}\mathrm{=3617}$, $k\mathrm{=0.52}$
*R*	0.5	0.1-1.5	optically thick medium, $R=O\mathrm{(1)}$ [22, 23]
*Q*	0.1	-	weak internal (e.g. absorbed) heating
*Nb*	0.1	0.1-0.4	Brownian-motion range of [32]
*Nt*	0.1	0.05-0.2	thermophoresis range of [32]
*Sc*	2	-	moderate species diffusivity
*K*, *n*, *E*	0.5, 0.5, 1	*K*: 0.5-2.0	Arrhenius reaction [74]
*δ *	0.1	-	${T}_{w}-{T}_{\infty }\approx 30$ K at ${T}_{\infty }\mathrm{=310}$ K, consistent with Eq. (15)
${\varphi }_{0}$	0.02	0-0.04	dilute gold loading

## Numerical procedure and validation

### Solution algorithm

Equations (19)-(22) with boundary conditions (23) form a two-point BVP on a semi-infinite domain. With ${y}_{1}=f$, ${y}_{2}=f{\prime}$, ${y}_{3}=f{\prime} {\prime}$, ${y}_{4}=g$, ${y}_{5}=g{\prime}$, ${y}_{6}=\theta$, ${y}_{7}=\theta {\prime}$, ${y}_{8}=\varphi$ and ${y}_{9}=\varphi {\prime}$, the problem is written as the first-order system

28 $$ \begin{array}{ll}{y}_{1{\prime} } & ={y}_{2},\,{y}_{2{\prime} }={y}_{3},\,{y}_{3{\prime} }=\frac{-{A}_{1}({y}_{1}{y}_{3}-{y}_{2}^{2})+\frac{{A}_{5}M}{1+{m}^{2}}({y}_{2}+m{y}_{4})-{A}_{6}Gr\,{y}_{6}-{A}_{7}Gc\,{y}_{8}}{{A}_{2}\mathrm{(1}+\mathrm{1/}\lambda )},\\ {y}_{4{\prime} } & ={y}_{5},\,{y}_{5{\prime} }=\frac{-{A}_{1}({y}_{1}{y}_{5}-{y}_{2}{y}_{4})-\frac{{A}_{5}M}{1+{m}^{2}}(m{y}_{2}-{y}_{4})}{{A}_{2}\mathrm{(1}+\mathrm{1/}\lambda )},\\ {y}_{6{\prime} } & ={y}_{7},\,{y}_{7{\prime} }=-\frac{Pr({A}_{3}{y}_{1}{y}_{7}+Nb\,{y}_{7}{y}_{9}+Nt\,{y}_{7}^{2}+Q\,{y}_{6})}{{A}_{4}+R},\\ {y}_{8{\prime} } & ={y}_{9},\,{y}_{9{\prime} }=-Sc\,{y}_{1}{y}_{9}-\frac{Nt}{Nb}\,{y}_{7{\prime} }+Sc\,K{\mathrm{(1}+\delta {y}_{6})}^{n}\exp (\frac{-E}{1+\delta {y}_{6}}){y}_{8},\end{array} $$

with $${y}_{1}\mathrm{(0)=0}$$, $${y}_{2}\mathrm{(0)=1}$$, $${y}_{4}\mathrm{(0)=0}$$, $${y}_{6}\mathrm{(0)=1}$$, $${y}_{8}\mathrm{(0)=1}$$ and $${y}_{2}({\eta }_{\infty })={y}_{4}({\eta }_{\infty })={y}_{6}({\eta }_{\infty })={y}_{8}({\eta }_{\infty }\mathrm{)=0}$$.

The system is solved with the fourth-order, three-stage Lobatto IIIA collocation method with residual control of Kierzenka and Shampine [75], the algorithm used by MATLAB's bvp4c [58]. In this work it was run through its SciPy implementation, solve_bvp [76]. The implementation details are:

- *Computational domain:*$0\le \eta \le {\eta }_{\infty }$ with ${\eta }_{\infty }\mathrm{=15}$, well beyond the momentum-layer edge ($f{\prime} {\mathrm{ < 10}}^{-2}$ at $\eta \mathrm{=8}$ in all cases, and $f{\prime}$ decays to zero well before ${\eta }_{\infty }$).
- *Mesh:* 400 uniformly spaced initial nodes, refined adaptively by the solver until the residual criterion is met (about 1600-1700 nodes at the baseline).
- *Initial guess:*$f\mathrm{=1}-{e}^{-\eta }$, $f{\prime} ={e}^{-\eta }$, $f{\prime} {\prime} =-{e}^{-\eta }$, $g=g{\prime} \mathrm{=0}$, $\theta ={e}^{-3\eta }$, $\theta {\prime} =-3{e}^{-3\eta }$, $\varphi ={e}^{-\eta }$, $\varphi {\prime} =-{e}^{-\eta }$, which satisfies all boundary conditions.
- *Tolerance and convergence criterion:* the relative collocation residual must be below ${10}^{-8}$ on every mesh interval, and the boundary conditions must be satisfied to the same tolerance. A run is accepted only when the solver reports convergence. All cases reported here converged directly from the above guess; otherwise the solution for a neighbouring parameter value would be used as the initial guess (continuation).

**Fig. 3:**
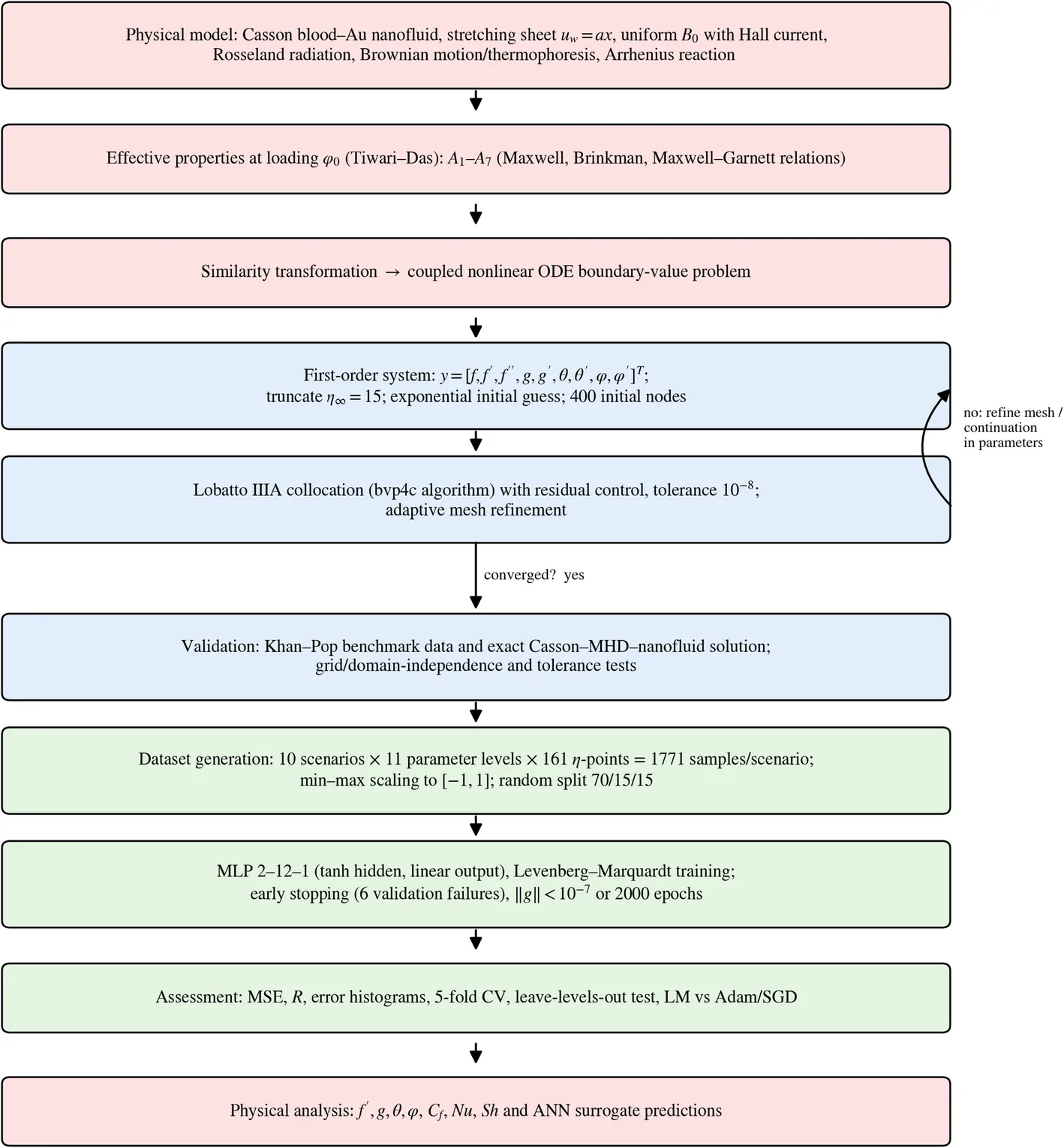
Flowchart of the solution algorithm: formulation, Lobatto IIIA (bvp4c-type) numerical solution with validation, dataset generation, Levenberg-Marquardt training and assessment of the ANN surrogate

The complete algorithm, including the ANN stage described in Section 4, is summarized in the flowchart of Fig. 3.

#### Grid and domain independence

Table 3 shows the wall quantities at the baseline for several domain lengths and tolerances. Changing ${\eta }_{\infty }$ from 12 to 20 or the tolerance from ${10}^{-6}$ to ${10}^{-10}$ leaves $f{\prime} {\prime} (0)$, $g{\prime} (0)$, $\theta {\prime} (0)$ and $\varphi {\prime} (0)$ unchanged to six decimals. Only $g{\prime} (0)$ differs, in the fifth decimal, for the shortest domain (${\eta }_{\infty }\mathrm{=8}$), which is why ${\eta }_{\infty }\mathrm{=15}$ is used throughout.

**Table 3:** Grid- and domain-independence test at the baseline parameters (Table 2)

${\eta }_{\infty }$	Tolerance	Final nodes	$f{\prime} {\prime} (0)$	$g{\prime} (0)$	$-\theta {\prime} (0)$	$-\varphi {\prime} (0)$	Max. residual
8	${10}^{-8}$	1611	*-0.541977*	0.097697	1.221862	0.610742	$1.0\times {10}^{-8}$
10	${10}^{-8}$	1656	*-0.541976*	0.097747	1.221861	0.610745	$1.0\times {10}^{-8}$
12	${10}^{-8}$	1681	*-0.541977*	0.097751	1.221861	0.610744	$1.0\times {10}^{-8}$
15	${10}^{-8}$	1689	*-0.541977*	0.097751	1.221861	0.610744	$1.0\times {10}^{-8}$
20	${10}^{-8}$	1622	*-0.541977*	0.097751	1.221861	0.610744	$1.0\times {10}^{-8}$
15	${10}^{-6}$	590	*-0.541977*	0.097751	1.221861	0.610744	$9.9\times {10}^{-7}$
15	${10}^{-10}$	6137	*-0.541977*	0.097751	1.221861	0.610744	$1.0\times {10}^{-10}$

### Code validation

The code is validated at three levels. (i) In the Newtonian, non-magnetic, buoyancy-free, pure-fluid limit with $Nb=Nt\mathrm{=0}$, the reduced Nusselt number $-\theta {\prime} (0)$ is compared with Khan and Pop [32] over $0.07\le Pr\le 70$ (Table 4); the largest difference is $2.7\times {10}^{-4}$, at $Pr\mathrm{=0.07}$, where Khan and Pop's own value differs by a similar amount from the earlier results they compared against. (ii) The Brownian-motion and thermophoresis coupling is checked against Khan and Pop's reduced Nusselt and Sherwood numbers for $Pr\mathrm{=10}$ and $Sc\mathrm{=10}$ (their Lewis number, defined as $\nu /{D}_{B}$); all twenty values in Table 5 agree to the four decimals reported. (iii) The Casson, MHD and nanofluid-property terms (which enter only through *λ *, *M* and ${A}_{1}$, ${A}_{2}$, ${A}_{5}$) are validated against an exact solution. For $m=Gr=Gc\mathrm{=0}$, Eq. (19) admits $f\mathrm{=(1}-{e}^{-\gamma \eta })/\gamma$ with

29 $$ f{\prime} {\prime} \mathrm{(0)=}-\gamma =-\sqrt{\frac{{A}_{1}+{A}_{5}M}{{A}_{2}(1+\mathrm{1/}\lambda )}}, $$

which reduces to Crane's result $f{\prime} {\prime} \mathrm{(0)=}-1$ [2] for a Newtonian pure fluid. Table 6 shows that the numerical and exact values agree to within ${10}^{-6}$ for Casson and Newtonian fluids with and without gold nanoparticles. Together these tests validate every term of the model except the Hall coupling, which is verified indirectly: *g≡ 0* is recovered for $m\mathrm{=0}$ (Table 10).

**Table 4:** Comparison of $-\theta {\prime} (0)$ with Khan and Pop [32] for $Nb=Nt\mathrm{=0}$, ${\varphi }_{0}\mathrm{=0}$, $\lambda \to \infty$, $M=Gr=Gc=R=Q=K\mathrm{=0}$

*Pr*	Khan and Pop [32]	Present	Absolute difference
0.07	0.0663	0.0666	$2.7\times {10}^{-4}$
0.2	0.1691	0.1691	$1.0\times {10}^{-5}$
0.7	0.4539	0.4539	$1.7\times {10}^{-5}$
2	0.9113	0.9114	$5.8\times {10}^{-5}$
7	1.8954	1.8954	$3.3\times {10}^{-6}$
20	3.3539	3.3539	$4.1\times {10}^{-6}$
70	6.4621	6.4622	$1.0\times {10}^{-4}$

**Table 5:** Comparison of the reduced Nusselt number $-\theta {\prime} (0)$ and Sherwood number $-\varphi {\prime} (0)$ with Khan and Pop [32] for $Pr=Sc\mathrm{=10}$ (other parameters as in Table 4)

*Nt*	*Nb*	$-\theta {\prime} (0)$ [32]	$-\theta {\prime} (0)$ present	$-\varphi {\prime} (0)$ [32]	$-\varphi {\prime} (0)$ present
0.1	0.1	0.9524	0.9524	2.1294	2.1294
0.1	0.2	0.5056	0.5056	2.3819	2.3819
0.1	0.3	0.2522	0.2522	2.4100	2.4100
0.1	0.4	0.1194	0.1194	2.3997	2.3997
0.1	0.5	0.0543	0.0543	2.3836	2.3836
0.5	0.1	0.3211	0.3211	3.0351	3.0351
0.5	0.2	0.1681	0.1681	2.8883	2.8883
0.5	0.3	0.0833	0.0833	2.7519	2.7519
0.5	0.4	0.0394	0.0394	2.6483	2.6483
0.5	0.5	0.0179	0.0179	2.5731	2.5731

**Table 6:** Validation of the Casson-MHD-nanofluid momentum solution against the exact result, Eq. (29) ($m=Gr=Gc\mathrm{=0}$)

*λ *	*M*	${\varphi }_{0}$	$f{\prime} {\prime} (0)$ exact	$f{\prime} {\prime} (0)$ numerical	Absolute difference
0.5	0	0	*-0.577350*	*-0.577351*	$1.0\times {10}^{-6}$
0.5	1	0.02	*-0.873728*	*-0.873728*	$6.1\times {10}^{-12}$
0.5	2	0.04	*-1.089720*	*-1.089720*	$1.3\times {10}^{-15}$
1.0	0	0	*-0.707107*	*-0.707107*	$9.4\times {10}^{-8}$
1.0	1	0.02	*-1.070094*	*-1.070094*	$2.6\times {10}^{-14}$
1.0	2	0.04	*-1.334629*	*-1.334629*	${\mathrm{ < 10}}^{-15}$
*∞ *	0	0	*-1.000000*	*-1.000000*	$3.8\times {10}^{-10}$
*∞ *	1	0.02	*-1.513341*	*-1.513341*	${\mathrm{ < 10}}^{-15}$
*∞ *	2	0.04	*-1.887451*	*-1.887451*	${\mathrm{ < 10}}^{-15}$

## Artificial neural network framework

### Data generation and preprocessing

The ANN is used as a surrogate that maps a physical parameter and the similarity coordinate to a solution profile. Ten scenarios (S1-S10, Table 7) are considered; in each, one parameter *p* is varied while all others are fixed at the baseline of Table 2. For every scenario, the validated solver of Section 3 is run at 11 equally spaced levels of *p* spanning the range in Table 7, and each converged profile is sampled at 161 equally spaced points on $\mathrm{[0,}{\eta }_{\max }]$. Here ${\eta }_{\max }\mathrm{=8}$ for $f{\prime}$, 2 for *θ * and 4 for *φ *, which covers the respective boundary layer ($Pr\mathrm{=21}$ makes the thermal layer thin). Each scenario therefore contains $11\times \mathrm{161=1771}$ input-target pairs, 17 710 in total. The inputs *(η ,p)* and the target are linearly scaled to $[-\mathrm{1,1]}$ (min-max normalization). Because *λ * enters the equations through $\mathrm{1/}\lambda$, $\mathrm{ln}\,\lambda$ is used as the input in S1. The samples are split at random into training (70%, 1240 samples), validation (15%, 266) and test (15%, 265) subsets. All errors are reported in physical (unscaled) units.

**Table 7:** Scenarios used to train and test the ANN; all other parameters are at the baseline values of Table 2

Scenario	Swept parameter *p*	Training levels (11, equally spaced)	Plotted cases	Output	Figure
S1	*λ *	0.2-2.0	0.2, 0.5, 1, 2	$f{\prime} (\eta )$	9
S2	*Gc*	0.5-2.0	0.5, 1, 1.5, 2	$f{\prime} (\eta )$	10
S3	*m*	0-1.5	0, 0.5, 1, 1.5	$f{\prime} (\eta )$	11
S4	*M*	0.5-3.0	0.5, 1, 2, 3	$f{\prime} (\eta )$	12
S5	*R*	0.1-1.5	0.1, 0.5, 1, 1.5	$\theta (\eta )$	13
S6	*Nb*	0.1-0.4	0.1, 0.2, 0.3, 0.4	$\theta (\eta )$	14
S7	*K*	0.5-2.0	0.5, 1, 1.5, 2	$\varphi (\eta )$	15
S8	*Nt*	0.05-0.2	0.05, 0.1, 0.15, 0.2	$\varphi (\eta )$	16
S9	${\varphi }_{0}$	0-0.04	0, 0.01, 0.02, 0.04	$f{\prime} (\eta )$	17
S10	${\varphi }_{0}$	0-0.04	0, 0.01, 0.02, 0.04	$\theta (\eta )$	18

### Network architecture and Levenberg-Marquardt training

**Fig. 4:**
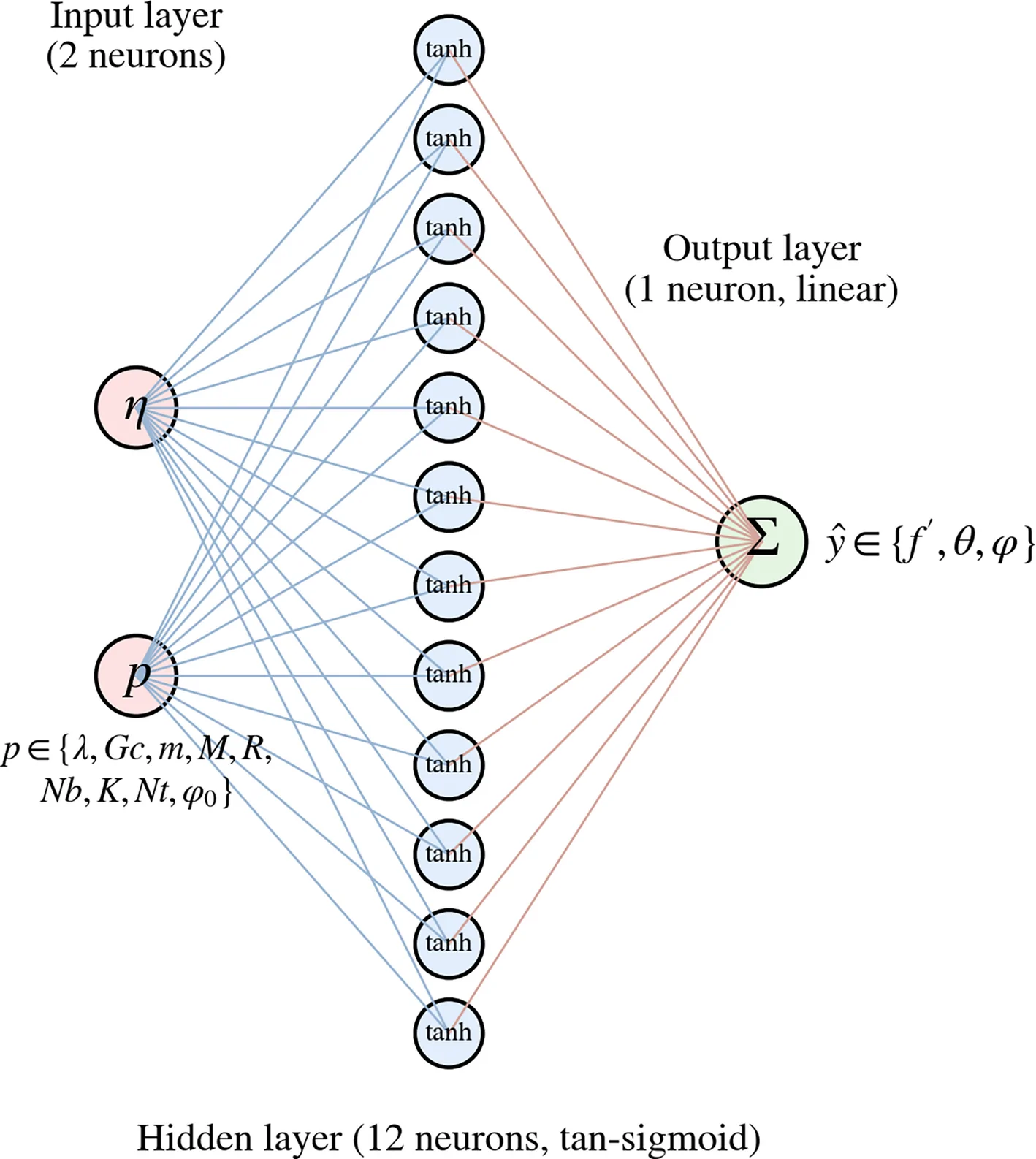
Architecture of the 2-12-1 multilayer perceptron: inputs *η * and the swept parameter *p*, 12 tan-sigmoid hidden neurons and a linear output neuron predicting $f{\prime}$, *θ * or *φ *

Each scenario uses a multilayer perceptron (Fig. 4) with two inputs *(η ,p)*, one hidden layer of 12 tan-sigmoid neurons and one linear output neuron, i.e. a 2-12-1 network with 49 weights and biases:

30 $$ \hat{y}(\eta ,p)={b}^{\mathrm{(2)}}+\mathop{\sum }\limits_{j\mathrm{=1}}^{12}{w}_{j}^{\mathrm{(2)}}\tanh \left({w}_{j1}^{\mathrm{(1)}}\eta +{w}_{j2}^{\mathrm{(1)}}p+{b}_{j}^{\mathrm{(1)}}\right)\mathrm{}. $$

Given the Jacobian ${\bf{J}}$ of the training errors ${\bf{e}}$ with respect to the weight vector ${\bf{w}}$, the LM algorithm [53, 54] updates the weights by

31 $$ \varDelta {\bf{w}}={({{\bf{J}}}^{T}{\bf{J}}+{\mu }_{{\rm{LM}}}{\bf{I}})}^{-1}{{\bf{J}}}^{T}{\bf{e}}\mathrm{}. $$

Here ${\mu }_{{\rm{LM}}}$ starts at ${10}^{-3}$, is multiplied by 0.1 after a successful step and by 10 after an unsuccessful one. The method therefore moves between Gauss-Newton (small ${\mu }_{{\rm{LM}}}$) and gradient descent (large ${\mu }_{{\rm{LM}}}$). Weights are initialized with the Nguyen-Widrow rule; three random initializations are trained and the one with the lowest validation error is kept. Training stops at the first of: 2000 epochs, a gradient norm below ${10}^{-7}$, ${\mu }_{{\rm{LM}}}{\mathrm{ > 10}}^{10}$, or six consecutive increases of the validation error (early stopping). The weights with the minimum validation error are returned. These settings are the defaults of MATLAB's trainlm; the network and training loop were implemented in Python/NumPy.

#### Why LM

For small networks trained on smooth, noise-free regression data, the LM method uses approximate second-order (curvature) information, which gives much faster and deeper convergence than first-order methods [54]. Its cost, one *49× 49* linear solve per iteration, is negligible here. Adaptive first-order methods such as Adam [77] and stochastic gradient descent are designed for large networks and data sets, where forming ${{\bf{J}}}^{T}{\bf{J}}$ is impractical. We tested this choice directly in Section 5.2.

#### Number of hidden layers and neurons

A single hidden layer suffices, since the targets are smooth functions of two variables. The number of neurons was chosen by training networks with 8, 12, 16 and 20 neurons on eight of the eleven parameter levels and testing them on the three held-out levels (the 3rd, 6th and 9th). The median held-out MSE over the ten scenarios was $5.6\times {10}^{-8}$, $8.8\times {10}^{-10}$, $1.8\times {10}^{-8}$ and $6.2\times {10}^{-9}$, and the worst maximum absolute error was $5.6\times {10}^{-3}$, $9.5\times {10}^{-4}$, $1.4\times {10}^{-2}$ and $8.7\times {10}^{-3}$, respectively. Networks with more neurons fit the training data more closely but interpolate less reliably between parameter levels, which is a sign of overfitting. Twelve neurons were therefore adopted.

#### Performance metrics

Accuracy is measured by the mean squared error ${\rm{MSE}}={N}^{-1}{\sum }_{i\mathrm{=1}}^{N}{({T}_{i}-{\hat{y}}_{i})}^{2}$, the correlation coefficient *R* between targets ${T}_{i}$ and outputs ${\hat{y}}_{i}$, the absolute error $|{T}_{i}-{\hat{y}}_{i}|$ and error histograms. Generalization is assessed by (i) 5-fold cross-validation, (ii) a leave-levels-out test in which the 3rd, 6th and 9th parameter levels are withheld entirely from training, and (iii) prediction of the plotted cases in Table 7 on a finer grid (321 points). Most of these plotted cases, e.g. $\lambda \mathrm{=0.5}$ and *1*, $M\mathrm{=1}$ and *2*, and ${\varphi }_{0}\mathrm{=0.01}$, are not among the training levels.

## Results and discussion

This section first assesses the neural surrogate (Figs. 5–8, Tables 8 and 9). It then discusses the physics of the velocity, temperature and concentration fields (Figs. 9–19) and of the wall quantities (Table 10, Fig. 20). In every profile figure, panel (a) compares the ANN prediction (coloured lines) with the numerical solution (dashed black lines), and panel (b) shows their absolute difference.

**Fig. 5:**
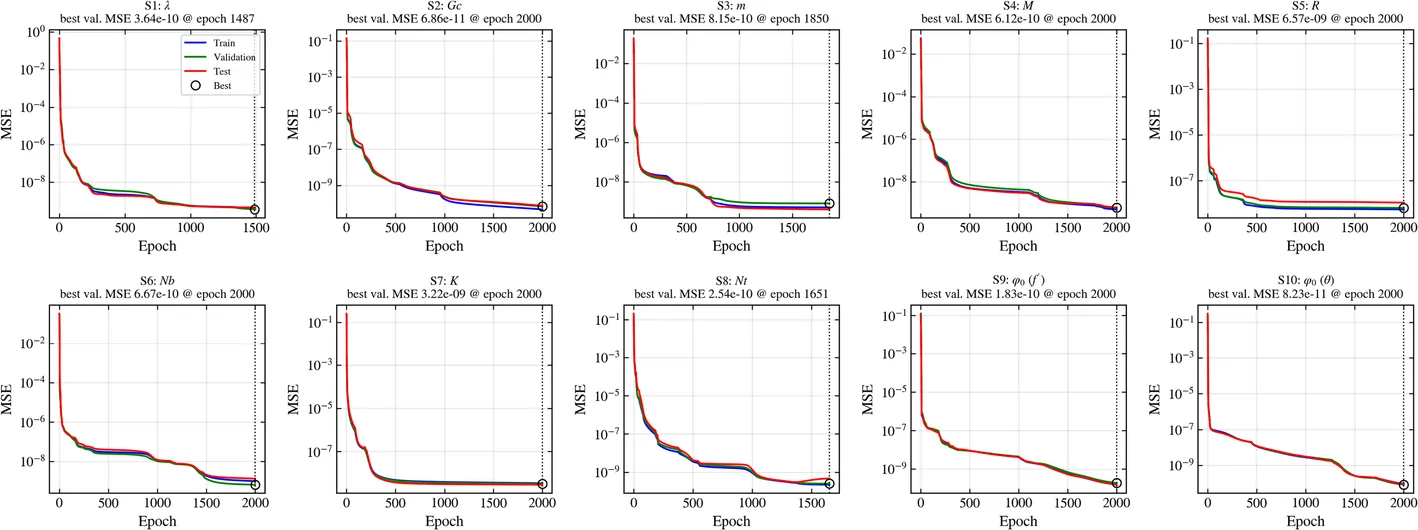
Training, validation and test MSE versus epoch for scenarios S1-S10; the circle marks the epoch with minimum validation error

**Fig. 6:**
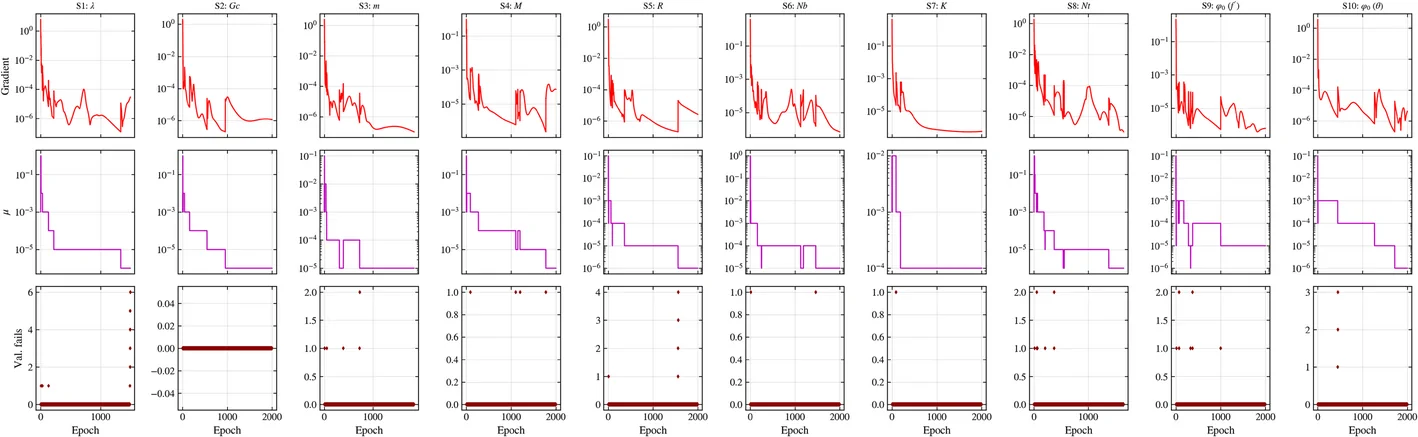
Training state for S1-S10: gradient norm (top), LM damping factor ${\mu }_{{\rm{LM}}}$ (middle) and number of consecutive validation failures (bottom)

**Fig. 7:**
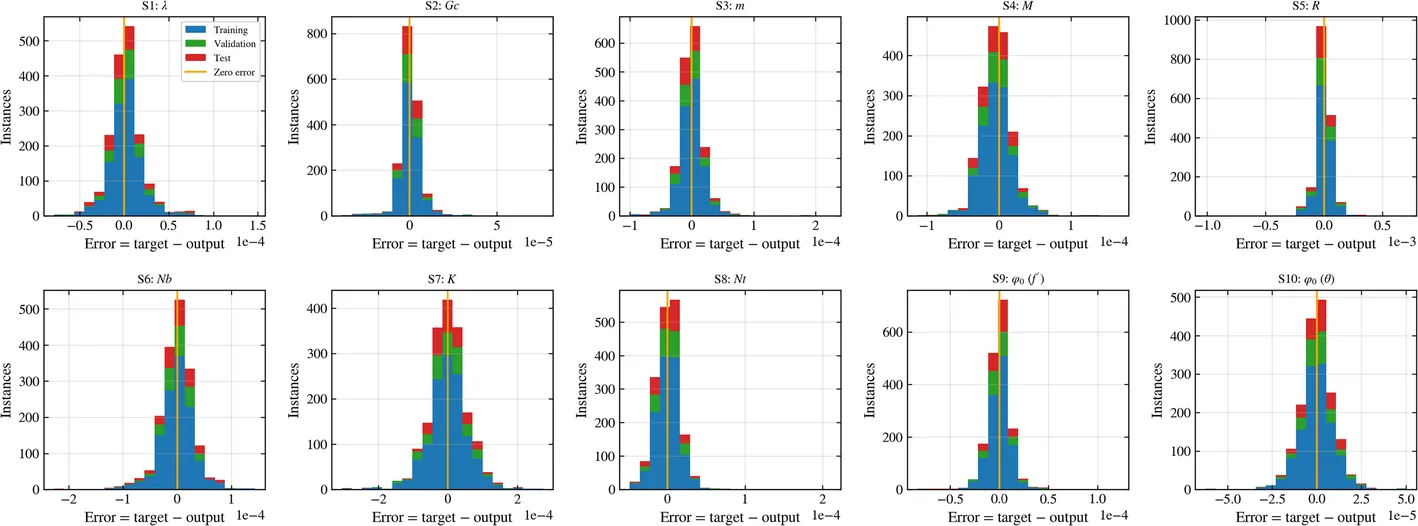
Error histograms (20 bins) of the training, validation and test samples for S1-S10

**Fig. 8:**
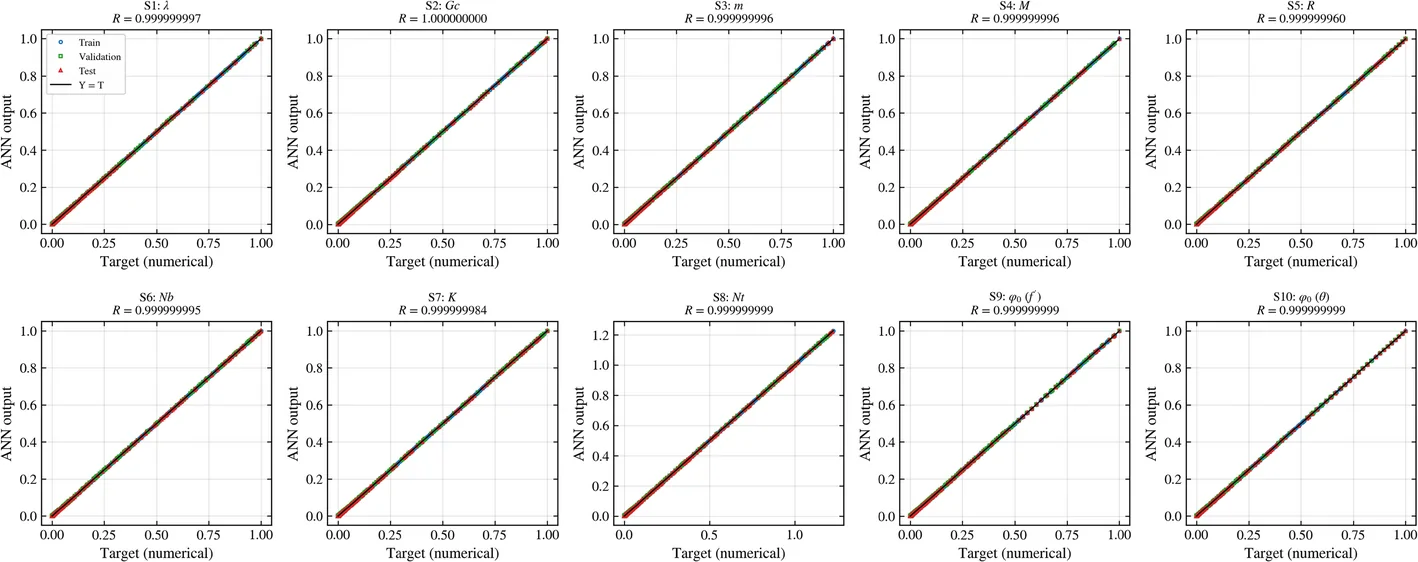
Regression of ANN outputs against numerical targets for S1-S10, with correlation coefficient *R*

**Table 8:** Performance of the LM-trained 2-12-1 networks (MSE in physical units)

Scenario	MSE			*R*	Gradient	${\mu }_{{\rm{LM}}}$	Best epoch	Stopping criterion	Time (s)
	Training	Validation	Testing						
S1	$3.85\times {10}^{-10}$	$3.64\times {10}^{-10}$	$4.58\times {10}^{-10}$	0.999999997	$2.5\times {10}^{-5}$	${10}^{-6}$	1487	validation stop	1.56
S2	$4.72\times {10}^{-11}$	$6.86\times {10}^{-11}$	$7.49\times {10}^{-11}$	1.000000000	$1.1\times {10}^{-6}$	${10}^{-6}$	2000	max. epochs	2.10
S3	$5.10\times {10}^{-10}$	$8.15\times {10}^{-10}$	$4.06\times {10}^{-10}$	0.999999996	$1.0\times {10}^{-7}$	${10}^{-5}$	1850	min. gradient	1.89
S4	$5.07\times {10}^{-10}$	$6.12\times {10}^{-10}$	$6.23\times {10}^{-10}$	0.999999996	$7.4\times {10}^{-5}$	${10}^{-6}$	2000	max. epochs	1.90
S5	$5.65\times {10}^{-9}$	$6.57\times {10}^{-9}$	$1.12\times {10}^{-8}$	0.999999960	$2.7\times {10}^{-6}$	${10}^{-6}$	2000	max. epochs	1.92
S6	$1.01\times {10}^{-9}$	$6.67\times {10}^{-10}$	$1.36\times {10}^{-9}$	0.999999995	$6.9\times {10}^{-7}$	${10}^{-5}$	2000	max. epochs	1.96
S7	$3.42\times {10}^{-9}$	$3.22\times {10}^{-9}$	$2.92\times {10}^{-9}$	0.999999984	$5.6\times {10}^{-7}$	${10}^{-4}$	2000	max. epochs	1.89
S8	$2.23\times {10}^{-10}$	$2.54\times {10}^{-10}$	$4.70\times {10}^{-10}$	0.999999999	$9.9\times {10}^{-8}$	${10}^{-6}$	1651	min. gradient	1.72
S9	$1.55\times {10}^{-10}$	$1.83\times {10}^{-10}$	$1.49\times {10}^{-10}$	0.999999999	$6.0\times {10}^{-7}$	${10}^{-5}$	2000	max. epochs	1.93
S10	$9.42\times {10}^{-11}$	$8.23\times {10}^{-11}$	$8.85\times {10}^{-11}$	0.999999999	$4.3\times {10}^{-6}$	${10}^{-6}$	2000	max. epochs	1.96

**Table 9:** Generalization tests and optimizer comparison

Scenario	5-fold CV MSE (mean*± *s.d.)	Leave-levels-out		Plotted cases	Test MSE (time, s)		
		MSE	max. error	max. error	LM	Adam	SGD
S1	$6.3\times {10}^{-8}\pm 9.6\times {10}^{-8}$	$1.6\times {10}^{-8}$	$9.5\times {10}^{-4}$	$1.5\times {10}^{-4}$	$4.6\times {10}^{-10}$(1.6)	$5.5\times {10}^{-6}$(2.0)	$6.0\times {10}^{-5}$(0.5)
S2	$1.5\times {10}^{-7}\pm 3.0\times {10}^{-7}$	$6.7\times {10}^{-10}$	$1.7\times {10}^{-4}$	$7.8\times {10}^{-5}$	$7.5\times {10}^{-11}$(2.1)	$8.6\times {10}^{-6}$(2.0)	$4.4\times {10}^{-5}$(0.5)
S3	$1.6\times {10}^{-9}\pm 5.5\times {10}^{-10}$	$2.4\times {10}^{-10}$	$8.1\times {10}^{-5}$	$1.9\times {10}^{-4}$	$4.1\times {10}^{-10}$(1.9)	$3.4\times {10}^{-6}$(2.0)	$3.3\times {10}^{-5}$(0.5)
S4	$7.9\times {10}^{-9}\pm 1.3\times {10}^{-8}$	$6.6\times {10}^{-9}$	$3.4\times {10}^{-4}$	$1.2\times {10}^{-4}$	$6.2\times {10}^{-10}$(1.9)	$7.5\times {10}^{-6}$(2.0)	$5.1\times {10}^{-5}$(0.5)
S5	$3.2\times {10}^{-8}\pm 5.2\times {10}^{-8}$	$6.4\times {10}^{-9}$	$2.0\times {10}^{-4}$	$1.0\times {10}^{-3}$	$1.1\times {10}^{-8}$(1.9)	$2.9\times {10}^{-6}$(1.9)	$4.7\times {10}^{-5}$(0.5)
S6	$1.5\times {10}^{-9}\pm 2.4\times {10}^{-9}$	$1.1\times {10}^{-9}$	$1.4\times {10}^{-4}$	$1.3\times {10}^{-4}$	$1.4\times {10}^{-9}$(2.0)	$1.2\times {10}^{-5}$(2.0)	$2.0\times {10}^{-5}$(0.5)
S7	$9.6\times {10}^{-6}\pm 1.9\times {10}^{-5}$	$1.8\times {10}^{-9}$	$2.7\times {10}^{-4}$	$3.1\times {10}^{-4}$	$2.9\times {10}^{-9}$(1.9)	$9.0\times {10}^{-5}$(1.9)	$1.9\times {10}^{-4}$(0.4)
S8	$5.6\times {10}^{-6}\pm 5.0\times {10}^{-6}$	$4.2\times {10}^{-10}$	$7.8\times {10}^{-5}$	$2.1\times {10}^{-4}$	$4.7\times {10}^{-10}$(1.7)	$8.4\times {10}^{-5}$(2.0)	$3.0\times {10}^{-4}$(0.6)
S9	$1.5\times {10}^{-7}\pm 2.1\times {10}^{-7}$	$3.5\times {10}^{-10}$	$1.2\times {10}^{-4}$	$1.2\times {10}^{-4}$	$1.5\times {10}^{-10}$(1.9)	$1.3\times {10}^{-6}$(1.9)	$2.2\times {10}^{-5}$(0.5)
S10	$1.1\times {10}^{-9}\pm 1.8\times {10}^{-9}$	$1.1\times {10}^{-11}$	$2.3\times {10}^{-5}$	$6.3\times {10}^{-5}$	$8.9\times {10}^{-11}$(2.0)	$7.2\times {10}^{-6}$(1.9)	$9.7\times {10}^{-6}$(0.5)

Test MSE (CPU time in s) is given for LM, Adam and SGD with identical network, initialization and split

**Fig. 9:**
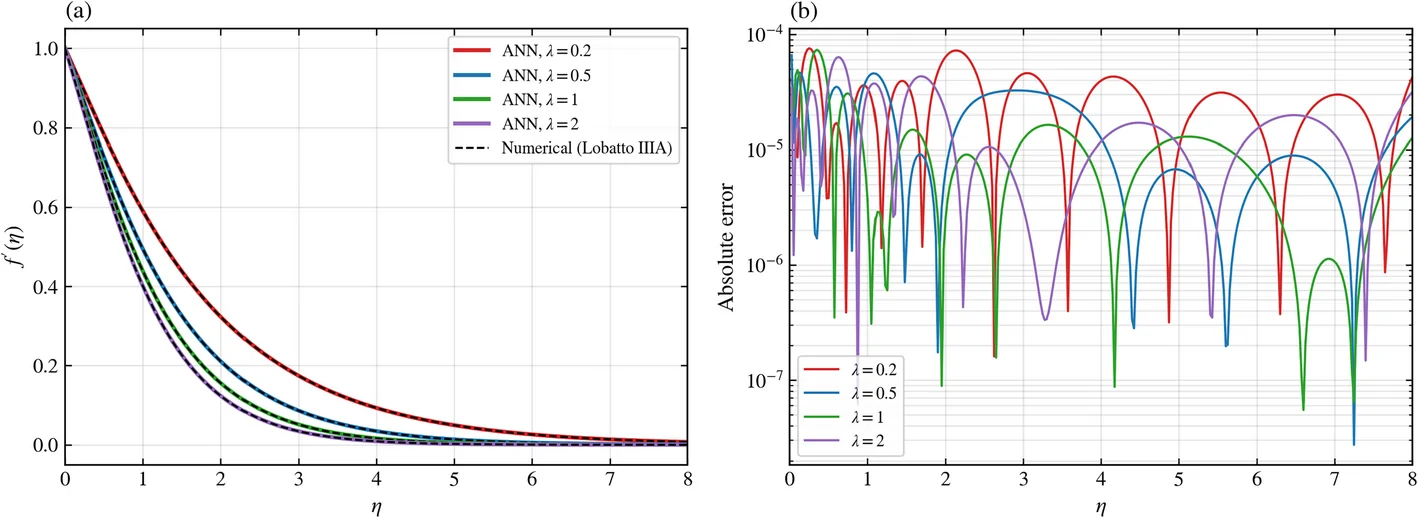
Effect of the Casson parameter *λ * on (**a**) $f{\prime} (\eta )$ and (**b**) ANN absolute error (S1; other parameters as in Table 2)

**Fig. 10:**
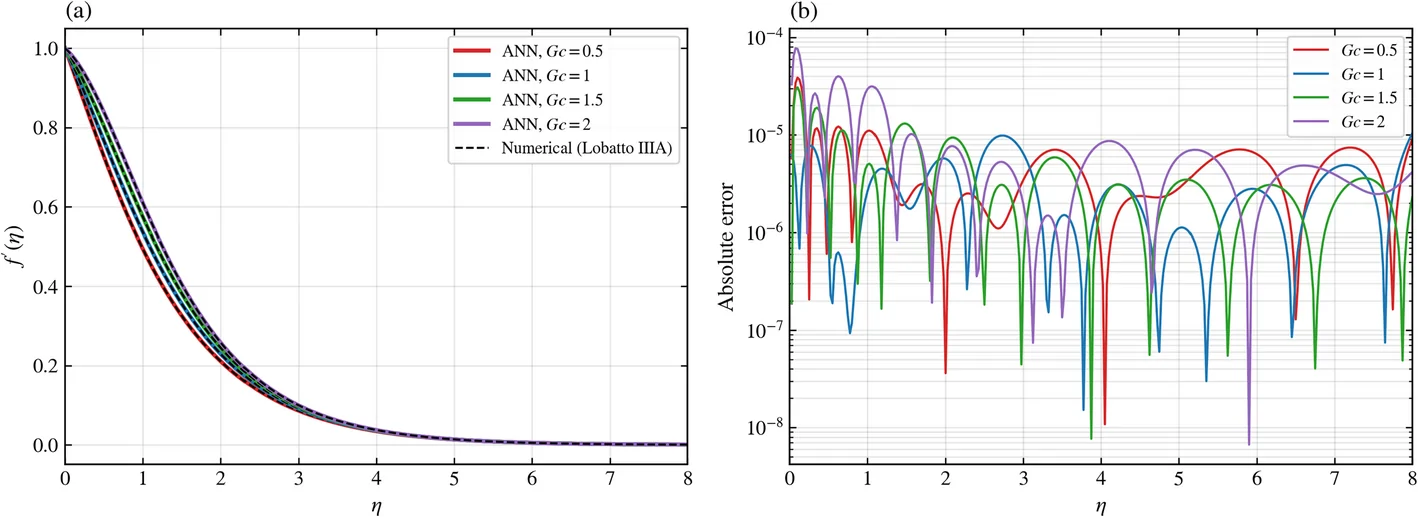
Effect of the solutal buoyancy parameter *Gc* on (**a**) $f{\prime} (\eta )$ and (**b**) ANN absolute error (S2)

**Fig. 11:**
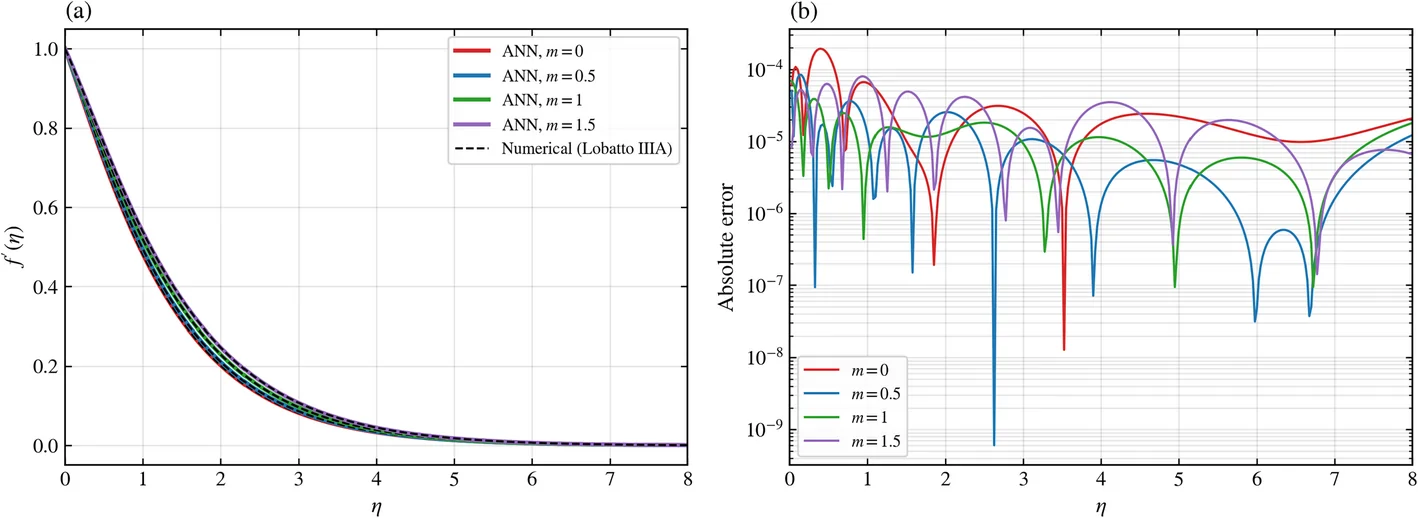
Effect of the Hall parameter *m* on (**a**) $f{\prime} (\eta )$ and (**b**) ANN absolute error (S3)

**Fig. 12:**
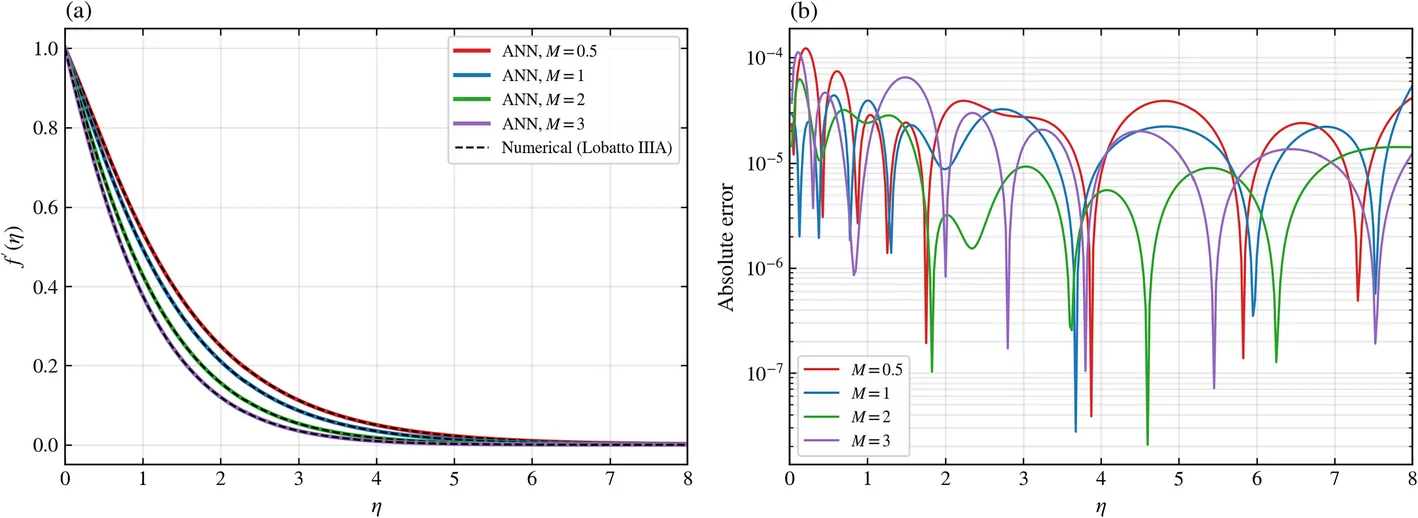
Effect of the magnetic parameter *M* on (**a**) $f{\prime} (\eta )$ and (**b**) ANN absolute error (S4)

**Fig. 13:**
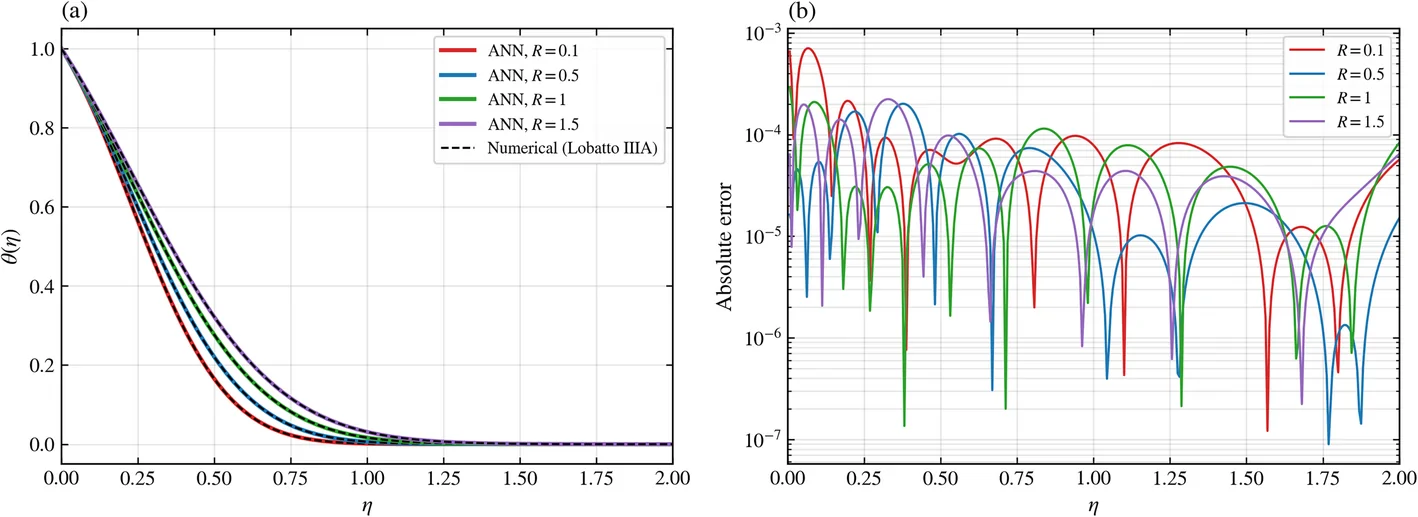
Effect of the radiation parameter *R* on (**a**) $\theta (\eta )$ and (**b**) ANN absolute error (S5)

**Fig. 14:**
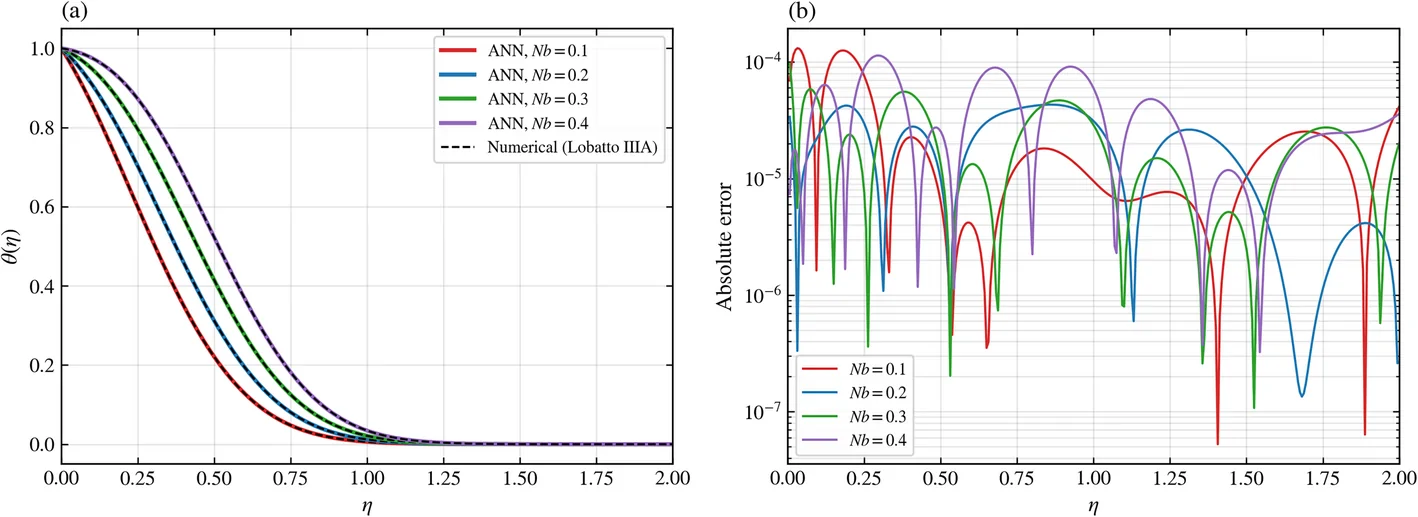
Effect of the Brownian-motion parameter *Nb* on (**a**) $\theta (\eta )$ and (**b**) ANN absolute error (S6)

**Fig. 15:**
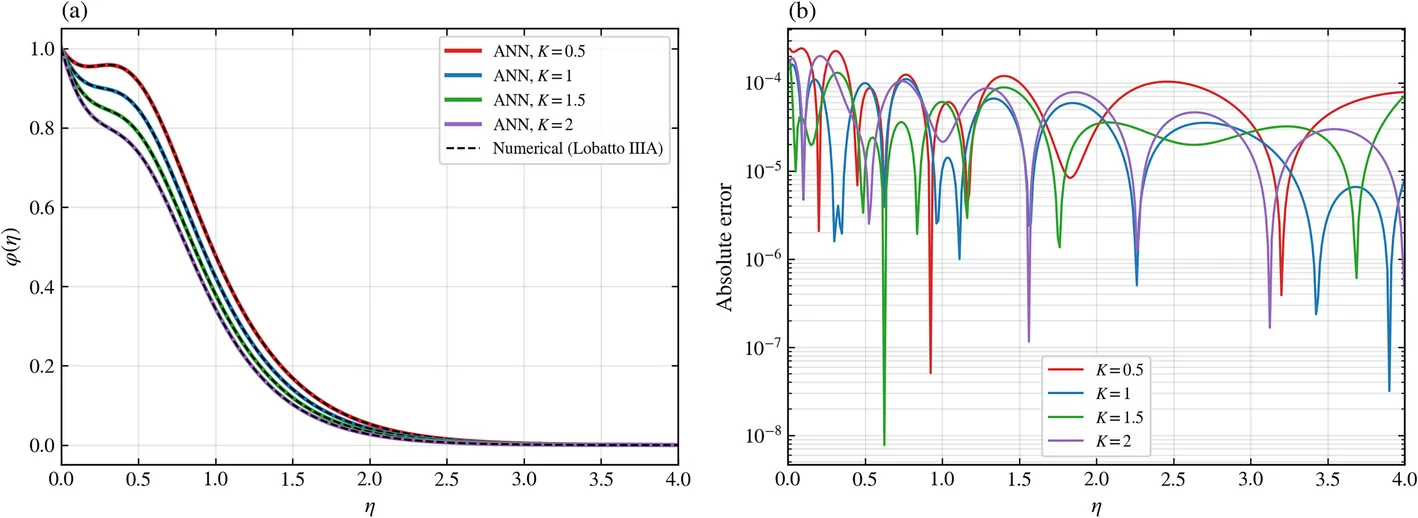
Effect of the chemical-reaction parameter *K* on (**a**) $\varphi (\eta )$ and (**b**) ANN absolute error (S7)

**Fig. 16:**
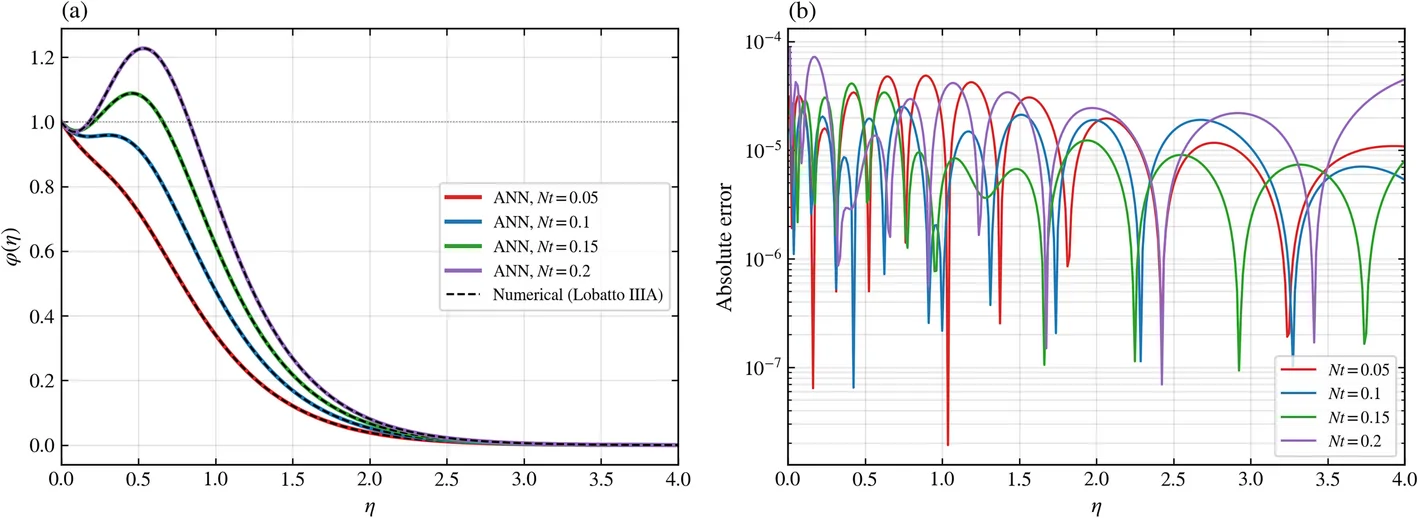
Effect of the thermophoresis parameter *Nt* on (**a**) $\varphi (\eta )$ and (**b**) ANN absolute error (S8); the dotted line marks $\varphi \mathrm{=1}$

**Fig. 17:**
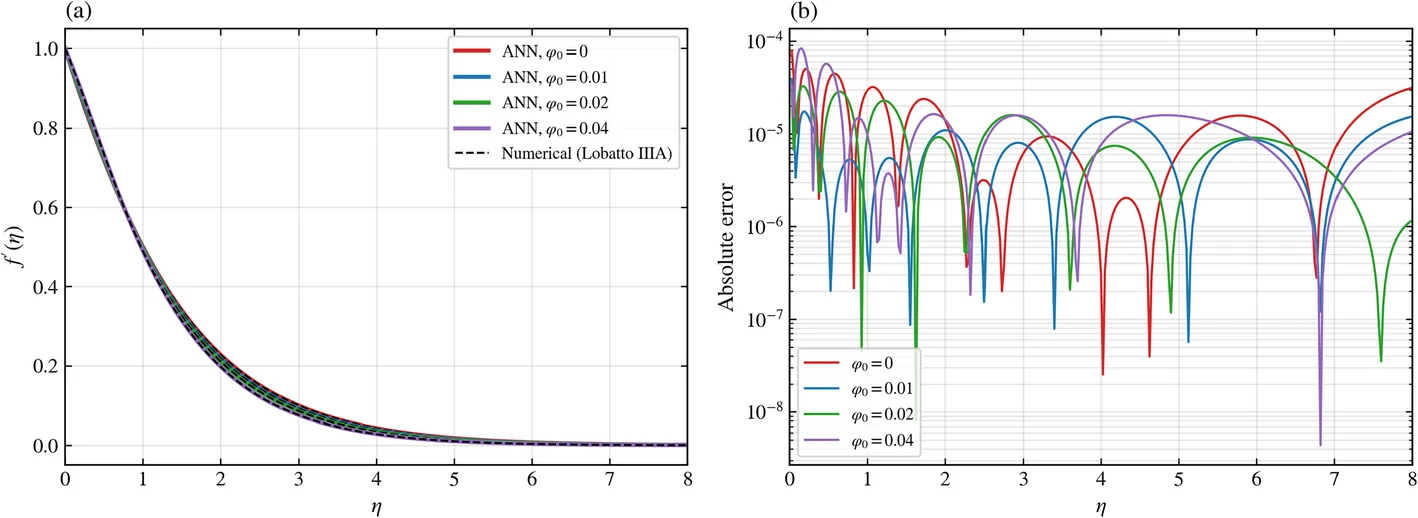
Effect of the gold volume fraction ${\varphi }_{0}$ on (**a**) $f{\prime} (\eta )$ and (**b**) ANN absolute error (S9)

**Fig. 18:**
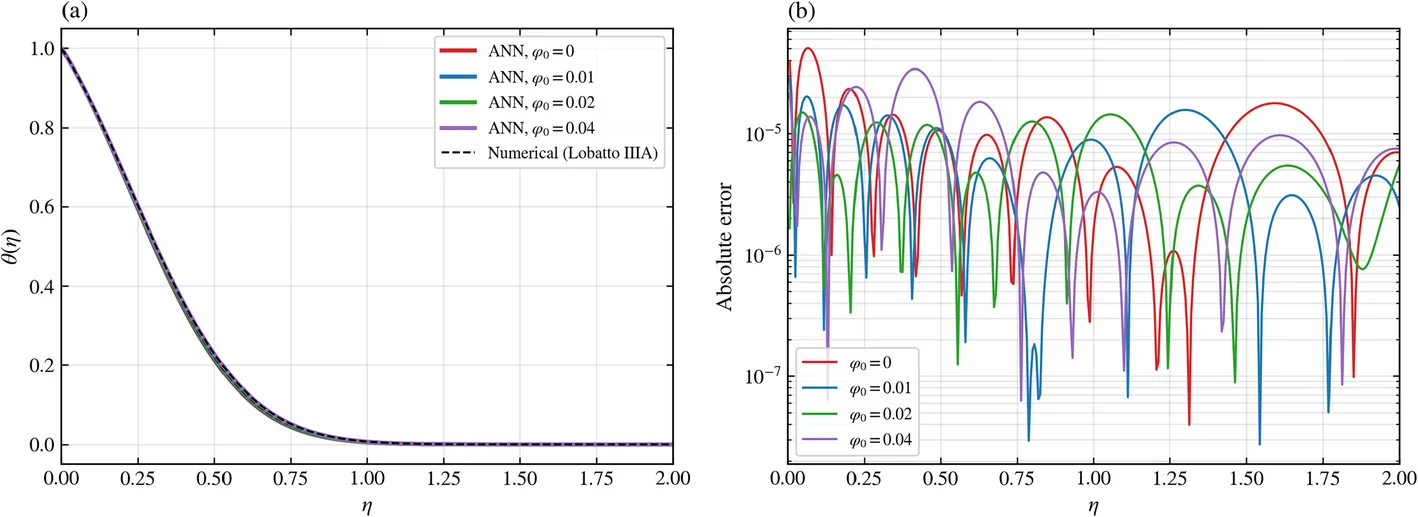
Effect of the gold volume fraction ${\varphi }_{0}$ on (**a**) $\theta (\eta )$ and (**b**) ANN absolute error (S10)

**Fig. 19:**
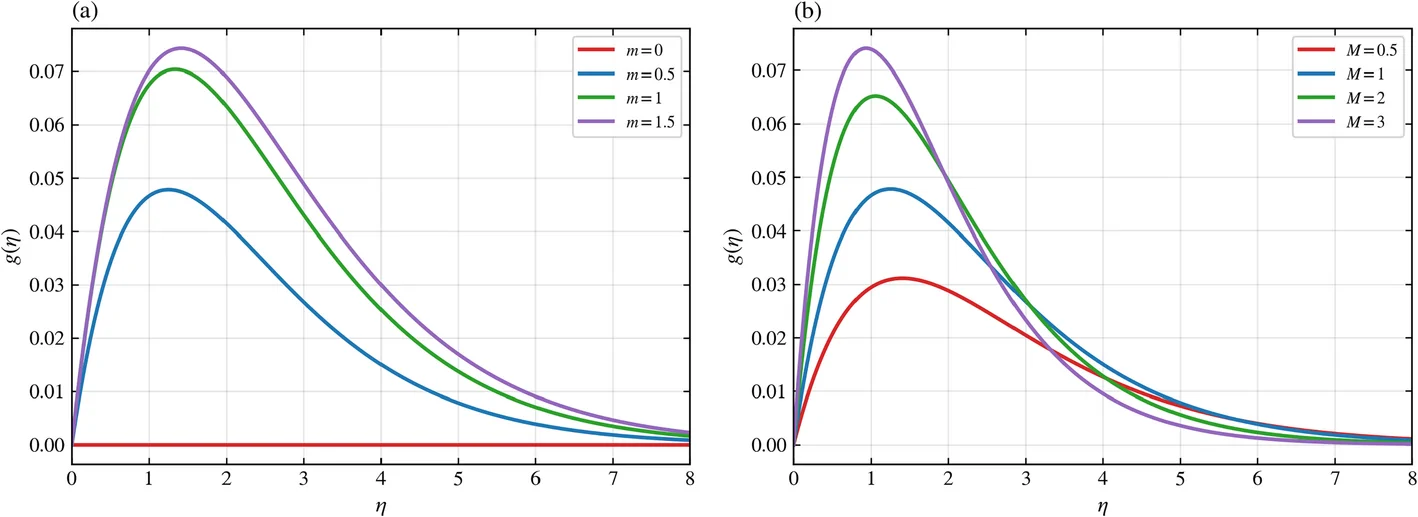
Hall-induced secondary velocity *g(η )* for (**a**) different *m* ($M\mathrm{=1}$) and (**b**) different *M* ($m\mathrm{=0.5}$)

**Table 10:** Skin-friction coefficients, Nusselt and Sherwood numbers, Eq. (27)

Parameter	Value	$R{e}_{x}^{{1/2}}{C}_{fx}$	$R{e}_{x}^{{1/2}}{C}_{fz}$	$R{e}_{x}^{-{1/2}}N{u}_{x}$	$R{e}_{x}^{-{1/2}}S{h}_{x}$
*λ *	0.2	*-2.7432*	0.4210	1.9140	0.6585
	0.5	*-1.7102*	0.3084	1.9072	0.6107
	1.0	*-1.2702*	0.2596	1.9036	0.5799
	2.0	*-1.0172*	0.2308	1.9012	0.5576
*Gc*	1.0	*-1.3110*	0.3218	1.9248	0.6311
	1.5	*-0.9256*	0.3337	1.9415	0.6491
	2.0	*-0.5515*	0.3445	1.9574	0.6652
*m*	0.0	*-1.8216*	0.0000	1.9027	0.6043
	1.0	*-1.5238*	0.4216	1.9143	0.6227
	1.5	*-1.3866*	0.4185	1.9192	0.6325
*M*	0.5	*-1.4249*	0.1776	1.9172	0.6317
	2.0	*-2.2152*	0.5038	1.8883	0.5751
	3.0	*-2.6570*	0.6532	1.8701	0.5460
*R*	0.1	*-1.7387*	0.3074	1.3999	0.6479
	1.0	*-1.6784*	0.3097	2.4702	0.6116
	1.5	*-1.6501*	0.3110	2.9730	0.6301
*Nb*	0.2	*-1.7110*	0.3068	1.0952	1.1168
	0.3	*-1.6580*	0.3078	0.5338	1.2364
	0.4	*-1.5937*	0.3096	0.1809	1.2662
*K*	1.0	*-1.7326*	0.3070	1.7782	0.9220
	1.5	*-1.7512*	0.3058	1.6748	1.1889
	2.0	*-1.7669*	0.3048	1.5897	1.4228
*Nt*	0.05	*-1.8079*	0.3041	2.2631	0.7779
	0.15	*-1.6193*	0.3127	1.5967	0.6075
	0.2	*-1.5353*	0.3167	1.3277	0.7293
${\varphi }_{0}$	0 (blood)	*-1.8805*	0.3005	1.8340	0.6107
	0.01	*-1.7949*	0.3042	1.8706	0.6105
	0.04	*-1.5434*	0.3185	1.9809	0.6123

Only the listed parameter differs from the baseline (Table 2); the baseline values are*-1.7102*, 0.3084, 1.9072 and 0.6107

**Fig. 20:**
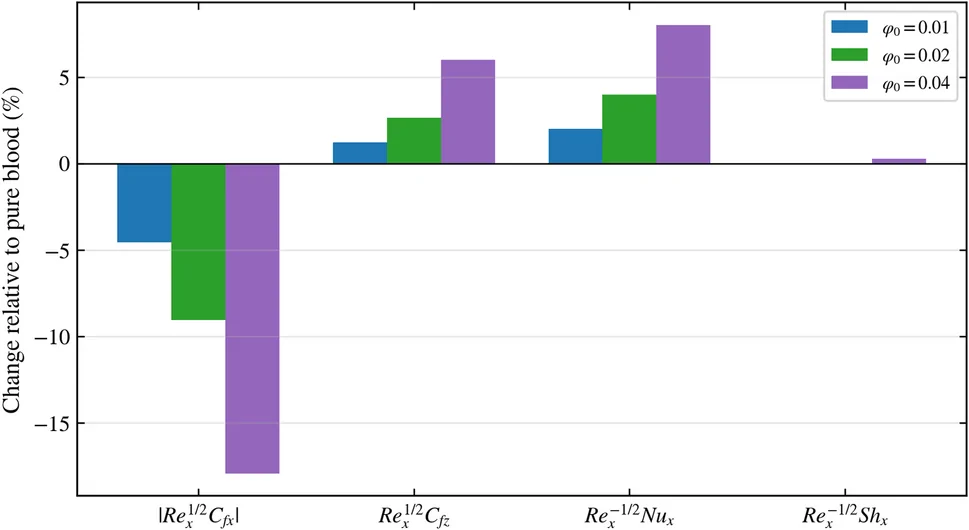
Percentage change of the wall quantities of the blood-Au nanofluid relative to pure blood (${\varphi }_{0}\mathrm{=0}$) at baseline conditions

### Performance of the ANN surrogate

Table 8 reports the training, validation and test MSE, the regression coefficient, the final gradient and damping factor, the best epoch, the stopping criterion and the CPU time for each scenario. The test MSE lies between $7.5\times {10}^{-11}$ (S2) and $1.1\times {10}^{-8}$ (S5), and *R* exceeds 0.99999996 in every case. The training, validation and test errors have the same order of magnitude in each scenario (e.g. $3.9\times {10}^{-10}$, $3.6\times {10}^{-10}$ and $4.6\times {10}^{-10}$ in S1), so the network fits unseen samples as well as training samples and shows no sign of overfitting. The performance curves (Fig. 5) fall by 7-9 orders of magnitude within the first few hundred epochs and then decrease slowly, with the three curves close together. The gradient and ${\mu }_{{\rm{LM}}}$ histories (Fig. 6) show the expected behaviour of the LM method: after an initial phase ${\mu }_{{\rm{LM}}}$ settles at ${10}^{-6}$-${10}^{-4}$, so the late iterations are essentially Gauss-Newton steps, and training ends by the gradient criterion (S3, S8), early stopping (S1) or the epoch limit. The error histograms (Fig. 7) are narrow and centred on zero, with errors of order ${10}^{-5}-{10}^{-4}$, and the regression plots (Fig. 8) lie on the line $\hat{y}=T$ for all subsets. One training run takes about 2 s on a single CPU core. After training, the network evaluates a full 321-point profile in about $2\times {10}^{-5}$ s, compared with about 0.15 s for one converged BVP solution, i.e. several thousand times faster. This speed-up matters when many evaluations are needed, e.g. in parameter sweeps, sensitivity analysis or embedded control, rather than for a single solution.

### Generalization, overfitting and choice of optimizer

Table 9 summarizes the generalization tests. The 5-fold cross-validated MSE is ${10}^{-9}-{10}^{-7}$ for eight scenarios. It is larger and more variable for the concentration scenarios S7 and S8 ($\sim {10}^{-5}$), where the profiles are steeper and, for S8, non-monotonic; this shows the sensitivity of a 49-weight network to the particular training subset. When whole parameter levels are withheld, the networks predict the missing profiles with MSE ${10}^{-11}-{10}^{-8}$ and maximum absolute errors below $9.5\times {10}^{-4}$. For the plotted cases, most of which are not training levels, the maximum absolute error is below $1.1\times {10}^{-3}$ (panel (b) of Figs. 9–18). The surrogate therefore interpolates reliably inside the trained ranges. It is not intended for extrapolation outside them (Section 6).

The last three columns of Table 9 compare LM with Adam [77] (full batch, learning rate $5\times {10}^{-3}$, 5000 epochs) and with SGD with momentum (mini-batch 64, learning rate ${10}^{-2}$, momentum 0.9, 500 epochs), using the same network, initial weights and data split and similar CPU time. LM gives a test MSE two to five orders of magnitude lower than Adam and 3.5 to 6 orders lower than SGD. This agrees with the known advantage of second-order methods for small, smooth regression problems [54] and supports the choice of LM for this surrogate.

### Velocity field

#### Casson parameter (Fig. 9)

Increasing *λ * from 0.2 to 2 lowers $f{\prime}$ throughout the layer ($f{\prime} \mathrm{(1)}$ falls from 0.590 to 0.401) and thins the momentum layer (the edge at which $f{\prime} \mathrm{=0.01}$ moves from $\eta \approx 7.5$ to 3.9). Since $\lambda ={\mu }_{B}\sqrt{2{\pi }_{c}}/{p}_{y}$, a larger *λ * means a smaller yield stress relative to the plastic viscosity, so the effective viscosity ${\mu }_{nf}\mathrm{(1}+\mathrm{1/}\lambda )$ decreases. Less momentum then diffuses from the moving sheet into the fluid, and the layer entrained by the sheet becomes thinner. The streamwise wall shear $|R{e}_{x}^{\mathrm{1/2}}{C}_{fx}|$ drops by 62.9% (Table 10). A more plastic (lower-*λ *) blood therefore drags a thicker fluid layer and needs a larger driving force.

#### Solutal buoyancy (Fig. 10)

Raising *Gc* from 0.5 to 2 raises $f{\prime}$ near the sheet ($f{\prime} \mathrm{(1)}$ from 0.495 to 0.610), because the concentration-driven buoyancy acts in the direction of the stretching (assisting flow) and adds a favourable body force inside the concentration layer. The wall shear decreases by 67.8%, because the buoyant fluid moves closer to the sheet speed and the sheet needs to supply less momentum.

#### Hall parameter (Fig. 11 and Fig. 19(a))

By Eq. (7), the streamwise Lorentz force is $-{\sigma }_{nf}{B}_{0}^{2}(u+mw\mathrm{)/(1}+{m}^{2})$. Increasing *m* reduces the effective conductivity ${\sigma }_{nf}\mathrm{/(1}+{m}^{2})$ in the primary direction, so $f{\prime}$ rises ($f{\prime} \mathrm{(1)}$ from 0.482 at $m\mathrm{=0}$ to 0.539 at $m\mathrm{=1.5}$) and $|{C}_{fx}|$ falls by 23.9%. At the same time the term *+mu* in the transverse force drives a secondary flow *g(η )*, which is absent for $m\mathrm{=0}$. Its peak grows from 0.048 $(m\mathrm{=0.5})$ to 0.074 $(m\mathrm{=1.5})$, and the transverse wall shear $R{e}_{x}^{\mathrm{1/2}}{C}_{fz}$ has a maximum near $m\mathrm{=1}$ (0.4216) before decreasing (0.4185 at $m\mathrm{=1.5}$). This non-monotonic response is characteristic of Hall MHD: the driving term $mM\mathrm{/(1}+{m}^{2})$ in Eq. (20) is largest at $m\mathrm{=1}$.

#### Magnetic parameter (Fig. 12 and Fig. 19(b))

Increasing *M* from 0.5 to 3 strengthens the retarding Lorentz force, which lowers $f{\prime} \mathrm{(1)}$ from 0.537 to 0.376 and thins the momentum layer from $\eta \approx 6.0$ to 4.0. The wall shear $|{C}_{fx}|$ rises by 86.5%. The same field also drives a stronger cross flow, whose peak grows from 0.031 to 0.074 and moves towards the wall (${\eta }_{\max }$ from 1.41 to 0.94); ${C}_{fz}$ increases 3.7-fold. In practice, the magnetic field acts as a non-contact brake on the near-wall blood layer. This is the mechanism behind magnetic flow control, but in blood it becomes significant only for strong fields and slow deformation rates (Section 2.4).

#### Gold loading (Figs. 17 and 20)

Adding gold has two competing effects on the velocity. The larger density (${A}_{1}$ from 1 to 1.70 at ${\varphi }_{0}\mathrm{=0.04}$) and viscosity $({A}_{2}=1.11)$ increase inertia and thin the momentum layer (edge from $\eta \approx 5.7$ to 5.05). At the same time the thermal-buoyancy ratio ${A}_{6}$ rises from 1 to 6.7, because gold has a much larger $\rho {\beta }_{T}$ than blood, and this accelerates the fluid inside the thin thermal layer. The result is a slightly faster near-wall flow and a slightly slower outer flow (Fig. 17), and a 17.9% reduction of $|{C}_{fx}|$ at ${\varphi }_{0}\mathrm{=0.04}$. The exact solution in Eq. (29) shows that, without buoyancy, gold would instead *increase* the wall shear: at $\lambda \mathrm{=0.5}$ and $M\mathrm{=1}$, $|f{\prime} {\prime} \mathrm{(0)|}$ rises from 0.816 for pure blood to 0.921 at ${\varphi }_{0}\mathrm{=0.04}$. The reduction of wall shear is therefore a buoyancy effect, and its size depends on the thermal-expansion data in Table 1.

### Temperature field

#### Thermal radiation (Fig. 13)

Increasing *R* from 0.1 to 1.5 raises $\theta \mathrm{(0.5)}$ from 0.165 to 0.322 and thickens the thermal layer ($\theta \mathrm{=0.01}$ edge from $\eta \approx 0.84$ to 1.19). In Eq. (21), *R* adds to the conductive diffusivity (${A}_{4}+R$), so radiation carries heat further from the sheet and lowers the dimensionless wall gradient $|\theta {\prime} \mathrm{(0)|}$. The total wall heat flux, which includes the radiative part, $R{e}_{x}^{-\mathrm{1/2}}N{u}_{x}=-({A}_{4}+R)\theta {\prime} (0)$, nevertheless increases by 112% (Table 10): radiation spreads the thermal energy over a thicker layer and increases the heat released from the surface. In photothermal settings this means stronger and deeper heating of the surrounding fluid.

#### Brownian motion (Fig. 14)

Raising *Nb* from 0.1 to 0.4 raises $\theta \mathrm{(0.5)}$ from 0.221 to 0.522. The term $Pr\,Nb\,\theta {\prime} \varphi {\prime}$ in Eq. (21) is positive, because both gradients are negative, and acts as a distributed heat source associated with the random motion of the particles. This source raises the fluid temperature near the wall and flattens the wall gradient, so the Nusselt number falls by 90.5%. Since $Pr\mathrm{=21}$ is large for blood, the Brownian contribution is strongly amplified. The accompanying 107% rise of the Sherwood number follows from the smaller thermophoretic counter-flux, whose relative weight *Nt/Nb* decreases as *Nb* increases.

#### Gold loading (Figs. 18 and 20)

The temperature rises slightly with ${\varphi }_{0}$ ($\theta \mathrm{(0.5)}$ from 0.213 to 0.228) because gold increases the thermal conductivity (${A}_{4}\mathrm{=1.124}$ at ${\varphi }_{0}\mathrm{=0.04}$) and hence the thermal diffusion. The wall heat flux increases by 4.0% at ${\varphi }_{0}\mathrm{=0.02}$ and by 8.0% at ${\varphi }_{0}\mathrm{=0.04}$ relative to pure blood, which quantifies the heat-transfer enhancement expected from gold's high conductivity. The Sherwood number is almost unchanged (+0.3%) because, in this model, ${\varphi }_{0}$ enters the concentration equation only through the velocity field.

### Concentration field

#### Chemical reaction (Fig. 15)

Increasing *K* from 0.5 to 2 lowers *φ * throughout the layer ($\varphi \mathrm{(0.5)}$ from 0.916 to 0.737) and thins the concentration layer, because the Arrhenius sink consumes particles more quickly as they diffuse away from the wall. The steeper wall gradient raises the Sherwood number by 133%. The Nusselt number falls by 16.6%, because a steeper $\varphi {\prime}$ strengthens the Brownian heating term $Nb\,\theta {\prime} \varphi {\prime}$ near the wall.

#### Thermophoresis (Fig. 16)

Thermophoresis drives particles from hot to cold regions, i.e. away from the heated sheet. With the wall concentration fixed, the outward thermophoretic flux collects particles just outside the wall. For *Nt≥ 0.15* this produces a concentration overshoot, ${\varphi }_{\max }\mathrm{=1.09}$ and 1.23 at $\eta \approx 0.46$ and 0.54 for $Nt\mathrm{=0.15}$ and 0.2. Such overshoots are a known feature of Buongiorno-type models when *Nt/Nb* exceeds about one and *Pr* is large, because the source term $(Nt/Nb)\theta {\prime} {\prime}$ in Eq. (22) is then large within the thin thermal layer. The same mechanism lowers the Nusselt number by 41.3% (*Nt* from 0.05 to 0.2) through the term $Pr\,Nt\,{\theta {\prime} }^{2}$. The Sherwood number varies non-monotonically (0.778, 0.611, 0.608, 0.729), because thermophoresis reverses the near-wall concentration gradient. The ANN reproduces the non-monotonic profiles with a maximum error of $2.1\times {10}^{-4}$.

### Skin friction, nusselt and sherwood numbers

Table 10 lists the four wall quantities of Eq. (27) for all scenarios, and Fig. 20 shows the change produced by the gold loading. The main parameters controlling each quantity over the ranges studied are:

- streamwise wall shear: *Gc* ($-\mathrm{67.8 \% }$), *λ * ($-\mathrm{62.9 \% }$) and *M* ($+\mathrm{86.5 \% }$), through buoyancy assistance, effective viscosity and Lorentz drag, respectively;
- transverse wall shear: *M* (3.7-fold) and *m* (maximum near $m\mathrm{=1}$), because the cross flow is driven only by the Hall term;
- Nusselt number: *R* ($+\mathrm{112 \% }$), *Nb* ($-\mathrm{90.5 \% }$) and *Nt* ($-\mathrm{41.3 \% }$); gold adds a moderate $+\mathrm{8.0 \% }$;
- Sherwood number: *K* ($+\mathrm{133 \% }$) and *Nb* ($+\mathrm{107 \% }$); *M* lowers it by 13.6% by reducing convective replenishment.

In general, the hydrodynamic parameters (*λ *, *M*, *m*, *Gc*) change the Nusselt number by less than 3%, because the thermal layer is much thinner than the momentum layer at $Pr\mathrm{=21}$. Heat transfer is controlled mainly by the thermal and particle-transport parameters.

### Practical implications and role of the ANN

The results give three practical guidelines for magnetically assisted nanofluid heat transfer. (i) The magnetic field and the Hall current regulate wall shear and redirect momentum into a cross flow without contact, but in blood a significant effect requires strong fields combined with slow deformation. (ii) Surface heat release is governed mainly by radiation and by the nanoparticle slip mechanisms, whereas a small gold loading gives a moderate (4-8%) gain. (iii) Surface reaction or binding strongly enhances mass transfer to the wall. These trends apply to the stretching-sheet configuration studied, e.g. coating, extrusion and polymer-processing flows and laboratory flows over deforming surfaces. They should be regarded as guidance on mechanisms, not as predictions for vessels or clinical devices (Section 6). The ANN adds a validated, smooth and very fast map from parameters to profiles. It can be queried at any intermediate parameter value, differentiated for sensitivity analysis, or embedded in optimization loops, without solving the BVP again.

## Limitations of the present study

1. *Idealized geometry*. The steady, laminar, two-dimensional stretching-sheet boundary layer does not represent a blood vessel, pulsatile flow, compliant walls or a therapeutic device. The results describe fundamental transport mechanisms, and any biomedical application (e.g. magnetic or photothermal therapy) would need vessel-scale models and experimental verification.
2. *Local similarity*. With a uniform wall temperature and concentration, *Gr* and *Gc* vary as ${x}^{-1}$, so the buoyancy-affected results are local similarity solutions.
3. *Electromagnetic parameters*. Order-one values of *M* in blood require strong fields and slow deformation rates, and the Hall parameter of an electrolyte is small; the *m*-range is parametric. Ion slip, induced fields and magnetization forces are neglected.
4. *Nanofluid model*. Effective properties from the Maxwell, Brinkman and mixing relations at a fixed loading ${\varphi }_{0}\le 0.04$ ignore particle size, aggregation, interfacial layers and haematocrit effects. The Tiwari-Das/Buongiorno combination assumes small concentration perturbations. The buoyancy effect of gold depends on the thermal-expansion data in Table 1.
5. *Radiation and reaction*. The linearized Rosseland model is valid only for optically thick media and small *δ *, and the Arrhenius sink is a generic representation of particle binding or reaction.
6. *ANN surrogate*. Each network is trained for one parameter at a time within the ranges of Table 7 and is reliable for interpolation only. Multi-parameter surrogates and extrapolation would need larger designs of experiments (e.g. Latin-hypercube sampling) and uncertainty quantification. The cross-validation spread for S7-S8 shows some sensitivity to the training subset.
7. *Validation*. Validation is against published numerical data and an exact solution; no experimental data exist for this configuration.

## Conclusions

A Casson blood-gold nanofluid flow over a stretching sheet with Hall current, radiation, Brownian motion, thermophoresis and an Arrhenius reaction was formulated in vector and boundary-layer form and reduced to a verified similarity system. It was solved by fourth-order Lobatto IIIA collocation and used to train Levenberg-Marquardt neural surrogates. The main findings are:

- The solver reproduces Khan and Pop's benchmark Nusselt and Sherwood numbers to four decimals and an exact Casson-MHD-nanofluid solution to within ${10}^{-6}$; the results are independent of the domain length and tolerance.
- Adding 4% gold to blood increases the Nusselt number by 8.0% (4.0% at 2%) and reduces the streamwise wall shear by 17.9%, the latter because gold strongly enhances the thermal buoyancy of the mixture.
- Increasing *M* from 0.5 to 3 raises the streamwise wall shear by 86.5% and the Hall-driven transverse shear 3.7-fold; the transverse shear peaks near $m\mathrm{=1}$, while increasing *m* from 0 to 1.5 lowers the streamwise shear by 23.9%.
- A larger Casson parameter (*λ *: 0.2*→ *2) reduces the effective viscosity, thins the momentum layer by about 47% and lowers the wall shear by 62.9%.
- Thermal radiation (*R*: 0.1*→ *1.5) thickens the thermal layer and increases the total wall heat flux by 112%, whereas Brownian motion and thermophoresis reduce the Nusselt number by 90.5% and 41.3%, respectively.
- The chemical reaction (*K*: 0.5*→ *2) raises the Sherwood number by 133%; thermophoresis with *Nt/Nb≥ 1.5* produces a near-wall concentration overshoot.
- The 2-12-1 LM networks reach test MSE of $7.5\times {10}^{-11}$-$1.1\times {10}^{-8}$ and $R\mathrm{ > 0.99999996}$, and interpolate to unseen parameter levels with errors below $1.1\times {10}^{-3}$. LM outperforms Adam and SGD by two to six orders of magnitude in test MSE at similar cost.

### Future work

Natural extensions are (i) multi-parameter surrogates trained on space-filling designs, with uncertainty quantification and physics-informed loss terms; (ii) unsteady and pulsatile flows in vessel-like geometries with compliant walls; (iii) two-phase or particle-size-dependent property models, ion slip and magnetization forces; (iv) non-linear radiation and entropy-generation analysis; and (v) comparison with experimental data for magnetically controlled nanofluid flows.

## Data Availability

The datasets used or analyzed during the current study are available from the corresponding author on reasonable request.

## Code availability

The solver and ANN training scripts used to generate all results are available from the corresponding author on reasonable request.

## Nomenclature

Latin symbols: ▓
*α*: stretching rate $$({s}^{-1})$$
*A*_1_−*A*_7_: nanofluid-to-blood property ratios, Eq. (21)
*B*_0_: applied magnetic induction (T)
*C*, *C*_*w*_, *C*_*∞*_: nanoparticle concentration; its wall and ambient values
*C*_*fx*_, *C*_*fz*_: skin-friction coefficients in the *x* and *z* directions
*c*_*p*_: specific heat $$({\rm{J}}\,{{\rm{kg}}}^{-1}\,{{\rm{K}}}^{-1})$$
*D*_*B*_, *D*_*T*_: Brownian and thermophoretic diffusion coefficients (m^2^ s^−1^)
*E*: dimensionless activation energy, ${E}_{a}/(\kappa {T}_{\infty })$
*E*_*a*_: activation energy (J)
*f*, *g*: dimensionless stream function and secondary velocity
*g*_*c*_: gravitational acceleration (m s^−2^)
*G*_*r*_, *G*_*c*_: thermal and solutal buoyancy (mixed-convection) parameters
**J**: current density (A m^−2^)
*k*: thermal conductivity (W m^−1^ K^−1^)
*k*^***^: Rosseland mean absorption coefficient (m^−1^)
${k}_{r}^{2}$: reaction-rate constant (s^−1^)
*K*: chemical-reaction parameter, ${k}_{r}^{2}/a$
*M*: magnetic parameter, $${\sigma }_{f}{B}_{0}^{2}/({\rho }_{f}a)$$
*m*: Hall parameter, $${\omega }_{e}{\tau }_{e}$$
*n*: temperature exponent of the reaction rate
*Nb*, *Nt*: Brownian-motion and thermophoresis parameters
*Nu*_*x*_, *Sh*_*x*_: local Nusselt and Sherwood numbers
*p*: pressure (Pa); also the swept physical parameter used as ANN input
*Pr*: Prandtl number, ${\nu }_{f}{(\rho {c}_{p})}_{f}/{k}_{f}$
*Q*, *Q*_0_: dimensionless and dimensional heat-source coefficients
*q*_*r*_, *q*_*w*_, *j*_*w*_: radiative heat flux, wall heat flux, wall mass flux
*R*: radiation parameter, $$16{\sigma }_{1}{T}_{\infty }^{3}\mathrm{/(3}{k}^{\ast }{k}_{f})$$
*Re*_*x*_: local Reynolds number, $a{x}^{2}/{\nu }_{f}$
*Sc*: Schmidt number, ${\nu }_{f}/{D}_{B}$
*T*, *T*_*w*_, *T*_*∞*_: temperature; its wall and ambient values (K)
*u*, *v*, *w*: velocity components along *x*, *y*, *z* (m s^−1^)
*U*_*w*_: sheet velocity, *ax*
*x*, *y*, *z*: Cartesian coordinates (m)
Greek symbols: ▓
*β*_*T*_, *β*_*C*_: thermal and solutal expansion coefficients
*δ*: temperature ratio, $$({T}_{w}-{T}_{\infty })/{T}_{\infty }$$
*η*: similarity variable
*θ*, *φ*: dimensionless temperature and concentration
*φ*_0_: gold nanoparticle volume fraction (loading)
*κ*: Boltzmann constant
*λ*: Casson parameter, $${\mu }_{B}\sqrt{2{\pi }_{c}}/{p}_{y}$$
*μ*, *μ*_*B*_: dynamic viscosity; plastic (Casson) viscosity (Pa s)
*μ*_LM_: Levenberg-Marquardt damping factor
*ν*: kinematic viscosity (m^2^ s^−1^)
*π*, *π*_*c*_: ${e}_{ij}{e}_{ij}$ and its critical value
*p*_*y*_: yield stress of the Casson fluid (Pa)
*ρ*: density (kg m^−3^)
*σ*: electrical conductivity (S m^−1^)
*σ*_1_: Stefan-Boltzmann constant
*τ*: heat-capacity ratio, ${(\rho c)}_{p}/(\rho {c}_{p}{)}_{f}$
*ψ*: stream function
*Subscripts and abbreviations*: ▓
*f*, *s*, *nf*: base fluid (blood), solid particle (gold), nanofluid
*w*, *∞*: wall and ambient conditions
ANN: artificial neural network
BVP: boundary-value problem
CV: cross-validation
LM: Levenberg-Marquardt
MHD: magnetohydrodynamics
MLP: multilayer perceptron
MSE: mean squared error
ODE/PDE: ordinary/partial differential equation
SGD: stochastic gradient descent
